# More spiritual than religious: Concurrent and longitudinal relations with personality traits, mystical experiences, and other individual characteristics

**DOI:** 10.3389/fpsyg.2022.1025938

**Published:** 2023-01-04

**Authors:** Zhuo Job Chen, Richard G. Cowden, Heinz Streib

**Affiliations:** ^1^School of Nursing, University of North Carolina, Charlotte, NC, United States; ^2^Human Flourishing Program, Institute for Quantitative Social Science, Harvard University, Cambridge, MA, United States; ^3^Research Center for Biographical Studies in Contemporary Religion, Bielefeld University, Bielefeld, Germany

**Keywords:** religion, spirituality, longitudinal, mysticism, more spiritual than religious

## Abstract

People who self-identify as predominantly spiritual constitute a considerable and well-established part of the religious landscape in North America and Europe. Thus, further research is needed to document predictors, correlates, and outcomes associated with self-identifying primarily as a spiritual person. In the following set of studies, we contribute to some of these areas using data from German and United States adults. Study 1 (*n* = 3,491) used cross-sectional data to compare four religious/spiritual (R/S) self-identity groups—more religious than spiritual (MRTS), more spiritual than religious (MSTR), equally religious and spiritual (ERAS), and neither religious nor spiritual (NRNS)—on sociodemographic characteristics and a range of criterion variables (i.e., Big Five personality traits, psychological well-being, generativity, mystical experiences, religious schemata). In Study 2 (*n* = 751), we applied the analytic template for outcome-wide longitudinal designs to examine associations of the four R/S self-identifications with a range of subsequent outcomes (assessed approximately 3 years later) that were largely comparable to the criterion variables assessed in Study 1. The cross-sectional and longitudinal findings from these complementary studies provide further evidence of differences between these four categories of R/S self-identification, including strong evidence in both studies of an association between the MSTR self-identity and mysticism.

## Introduction

Since spirituality escaped the walls of the monasteries and the niches of ‘spiritual discipline,’ it has given rise to the rather widespread popular self-identification of ‘I am spiritual’ that presents the scientific study of religion with new conceptual puzzles and empirical challenges. When spirituality indicates changes from traditional religious beliefs or introduces beliefs that were *not* previously considered part of established religious traditions, new developments in the field of religious studies may emerge from research involving people with a preference for spirituality. For example, spirituality has been interpreted as a new search for the sacred, including processes related to the sanctification of worldly objects (Pargament, 1997; Pargament and Mahoney, 2017); spirituality may represent a new understanding of what constitutes being ‘ultimately concerned’ (Tillich, 1957; Streib and Hood, 2016b); or spirituality may be associated with a dimension of transcendence that focuses on targets within this world, such as nature, humanity, or the universe, rather than supernatural beings. This dimension of transcendence may be labeled ‘horizontal,’ which contrasts the ‘vertical’ (primarily theistic) dimension of transcendence that is more typical of religious worldviews (Schnell, 2012; Mercadante, 2014; Streib and Hood, 2016b). These features of spirituality are most common among people who identify as ‘spiritual but not religious’ (SBNR), a group that may represent a post-Christian form of spirituality (Houtman and Aupers, 2007; Houtman and Tromp, 2020).

Of course, not everyone who includes spirituality in their self-identification insists on such a sharp contrast. There are different perspectives on whether religion and spirituality are overlapping or even identical concepts, or whether they are in opposition to each other. The SBNR self-identification is based on an opposition, but there are people who identify as ‘religious but not spiritual’ (nevertheless, it looks like carrying owls to Athens to propose this today, see Simmons, 2021). However, we should not ignore that there is also widespread conviction of an overlap between religion and spirituality. This is presented by two groups that equally either identify with, or reject, both religion and spirituality. Therefore, researchers are well advised to distinguish between at least four categories of religious/spiritual (R/S) self-identification. In order to avoid excluding individuals who do not see a sharp contrast or mutual exclusivity between religion and spirituality, researchers have begun to use ‘more than’ phrasing to group individuals into one of four R/S identities (see Hood, 2003; Streib et al., 2009, 2016a, 2022; Streib and Klein, 2018): ‘more religious than spiritual’ (MRTS), ‘more spiritual than religious’ (MSTR), ‘equally religious and spiritual’ (ERAS), and ‘neither religious nor spiritual’ (NRNS). To strengthen our understanding of similarities and differences between R/S identities, in the current set of studies we use this categorization approach to explore concurrent (Study 1) and prospective (Study 2) associations of these four R/S identities with a wide range of variables.

### Subjective definitions of spirituality are multiplicitous

Coming to terms with a definition of spirituality and its relation to religion on the conceptual level is a challenge and an unsolved problem; indeed, definitions of spirituality vary (Oman, 2013). Using a content-analytical approach to explore the concept of spirituality, Harris et al. (2018) documented a broad spectrum of categories reflected in the term. Similarly, in their research on people who identify as SBNR, Wixwat and Saucier (2021, p. 124) concluded that “spirituality is itself heterogeneous,” and that there are “multiple meanings for spirituality” involved in the SBNR self-identification. Even among those who identify as SBNR, there is considerable diversity, such as in religious affiliation, R/S practices, and importance of religion (Tong and Yang, 2018), and the SBNR identification itself may be subject to change over time (Upenieks and Ford-Robertson, 2022).

The multiplicity of meanings of spirituality in the SBNR and MSTR has been confirmed and further detailed in numerous studies that have applied an emic approach to investigate participants’ own definitions of religion and spirituality (e.g., Zinnbauer et al., 1997; Hyman and Handal, 2006; Schlehofer et al., 2008; la Cour et al., 2012; Ammerman, 2013; Berghuijs et al., 2013; Eisenmann et al., 2016; Steensland et al., 2018; Demmrich and Huber, 2020). Importantly, these studies have revealed, perhaps counterintuitively, that a great number of highly spiritual participants assume an overlap or shared identity between religion and spirituality and describe spirituality in traditional religious language. For example, Ammerman’s (2013) qualitative research with a diverse sample of United States adults prompted her conclude that the assumption of a ‘binary’ either/or contrast between organized religion and spirituality should be avoided, since it may lead to a misunderstanding of the spirituality of those who identify as SBNR. She suggested that spirituality falls into four categories: (1) theistic, (2) extra-theistic, (3) ethical, and (4) belief and belonging. In another study of United States adults, Steensland et al. (2018) identified seven latent classes from 1,038 responses to an open-ended question that asked participants to describe what spirituality meant to them: (1) organized religion, (2) belief in God, (3) relationship with God, (4) belief in a higher being, (5) belief in something beyond, (6) holistic connection, and (7) ethical action. These examples, among others, suggest that multiplicitous subjective meanings of spirituality can be classified under three broad banners: (1) a more ‘religious’ understanding that associates spirituality with God, Jesus, the Bible, organization, and a system; (2) an understanding of spirituality as connectedness with other humans, the inner or higher self, nature, and the universe, which eventually relates to mysticism; and (3) an understanding that relates spirituality to morality/ethics and an orientation toward humanity. To complement the rich qualitative evidence on subjective understandings of religion and spirituality, additional interpretative insights about R/S identities may emerge from examining their associations with individual characteristics and relevant outcomes.

### Differentiating MSTR and SBNR identities from other R/S identities

There is a relatively small (but growing) number of studies that have focused on the correlates of self-identifying as MSTR or SBNR with personality characteristics, indices of well-being, and mystical experiences (among other factors), with some research identifying predictors and outcomes for the MSTR self-identification. Interesting research questions and assumptions have emerged from the existing empirical literature, although some evidence is more mixed and requires further attention.

#### Cognitive structures, supernatural beliefs and experiences, and spirituality

Willard and Norenzayan’s (2017) cross-sectional study of United States adults was a key step in profiling the cognitive bias and beliefs of people who identify as SBNR. They compared three groups (i.e., SBNR, religious, nonreligious) on cognitive characteristics (e.g., mentalizing/empathy quotient), supernatural beliefs (e.g., belief in God), mystical experiences (e.g., universal connectedness), and schizotypy measures (e.g., unusual perceptual experiences). They found that the cognitive profile of the SBNR group was similar to the religious group, except for mind–body dualism which was higher in the religious group. However, the SBNR group differed from the conventionally religious group in that those participants appeared “more prone to paranormal beliefs, are more likely to have an experiential relationship to the supernatural, and see themselves more connected to the universe as a whole” (Willard and Norenzayan, 2017, p. 144). The SBNR group also scored higher than the religious and nonreligious groups on several schizotypy measures (e.g., magical ideation, unusual perceptions), demonstrating their proclivity to schizotypal experiences.

In a more recent cross-sectional study, Lindeman et al. (2019) have identified and compared three latent class groups in multiple European countries: (1) analytic atheists, (2) SBNR, and (3) uncertain non-believers. Although the SBNR group evidenced strong disagreement with belief in God (thus, the SBNR group in their study is not likely to have included many spiritual individuals who believed in God), Lindeman et al. (2019) suggested that “belief in supernatural phenomena explains most of the variance in self-reported spirituality” (p. 197). Therefore, they considered their findings as being in line with previous studies that have found people who identify as SBNR tend to view “the human mind more as a porous entity than a bounded entity, which implies the possibility of direct communication with the supernatural (e.g., hearing voices of supernatural agents, etc.)” (Lindeman et al., 2019, p. 197). They also noted that the SBNR participants “were lowest and strongly religious individuals were highest on the dimension that included only traditional values” (p. 198).

These abovementioned studies point to the possibility of uncovering new insights by exploring people who identify as SBNR, which may eventually lead to hypotheses about the content of their supernatural beliefs (with special attention to paranormal beliefs), their inclination to experience hallucinations and magical thinking, and their preferences for experience (including mysticism and connectedness). Although our ability to explore these areas in the current set of studies is somewhat constrained by the limits of the data that are available to us, we are able to test some assumptions within the experiential dimension (e.g., mysticism).

#### Personality traits and spirituality

Previous studies generally suggest that self-rated spirituality is related to higher scores on openness to experience, but findings for the other four of the Big Five personality traits are more mixed. MacDonald’s (2000) cross-sectional research with Canadian undergraduate students documented evidence of associations between openness to experience and dimensions of spirituality, especially the cognitive, experiential, and paranormal belief dimensions, but not for the religiousness dimension. In one study of United States adults that distinguished tradition-oriented religiousness from subjective spirituality, Saucier and Skrzypińska (2006) found that subjective spirituality was positively correlated with openness to experience, whereas tradition-oriented religiosity correlated negatively with openness to experience and positively with agreeableness. Using a two-wave panel design with a sample of United States adolescents who were followed through to late adulthood, Wink et al. (2007) found that openness to experience in adolescence was positively associated with spiritual seeking in late adulthood, whereas conscientiousness in adolescence was positively associated with religiousness in late adulthood. In a cross-sectional study of Spanish university students, Saroglou and Muñoz-García (2008) found that both spirituality and religiosity were positively correlated with agreeableness and consciousness, but only spirituality correlated positively with openness to experience and only religiosity correlated positively with neuroticism. Finding that their results align with Saroglou’s (2002) earlier meta-analysis, Saroglou and Muñoz-García (2008) suggested that “spiritual people find in spirituality some elements that allow them to maintain a sense of self control … especially given the presence of some neurotic tendencies (similarly to religion) in the present sample. However, they seem able to use new and autonomous ways to deal with experience and spiritual meaning” (p. 98). In a more recent meta-analysis of research on personality and religion, which included 71 studies from 19 countries and more than 20,000 participants in total, Saroglou (2010) reported that spirituality, religiosity, and religious fundamentalism were all positively correlated with agreeableness and conscientiousness. But spirituality/mature faith correlated positively with openness to experience and extraversion, whereas religious fundamentalism correlated negatively with openness to experience. Religiosity was unrelated to the other three Big Five personality traits.

Other studies have addressed associations of spiritual self-identity with and without religious identity. For example, in a cross-sectional study of first-year undergraduate students in Austria, Schnell (2012) explored differences in the Big Five personality traits between individuals who were spiritual and those who were both spiritual and religious. There was evidence of higher agreeableness in the latter group, but no other differences emerged. Schnell (2012, p. 56) suggested that “the spiritual-but-not-religious are not more open than the religious-and-spiritual,” although “both types show a very high openness compared to the general population.” She also asserted that openness to experience is “fundamentally close to spirituality, on factor and facet level. A high openness for ideas, aesthetics and feelings is evident” (p. 53). More recently, Streib et al. (2016) used adult United States and German samples to examine differences between four R/S self-identities (i.e., MRTS, MSTR, ERAS, NRNS) on the Big Five personality traits. The MSTR and NRNS groups in both the United States and German samples scored particularly high on openness to experience, and the means for these groups were higher than normative values in each country.

Overall, evidence accumulated so far generally supports the assumption that self-attributed spirituality is associated with the Big Five personality trait of openness to experience. There is some evidence that agreeableness (and to some extent conscientiousness) is associated with religion, whereas openness to experience relates positively to self-rated spirituality and negatively with religiousness (see Wixwat and Saucier, 2021). However, much of the existing literature in this area is based on cross-sectional data, and therefore evidence that addresses causal linkages between personality traits and spirituality is relatively limited.

#### Psychological well-being and spirituality

Many studies have reported on the relation between spirituality and psychological well-being, but most of these studies either use religion and spirituality interchangeably or use measurements that confound spirituality and psychological well-being, and most are based on cross-sectional samples (see also the critique by Highland et al., 2022). When we focus on studies that differentiate between groups that reflect dominant R/S identities (i.e., MRTS, ERAS, MSTR, NRNS) and on studies that do not confound spirituality and psychological well-being, a relatively small number remains. However, these studies often differ in how they measure spirituality, and their findings have been mixed.

Nadal et al. (2018) investigated psychosocial functioning in a cross-sectional sample of emerging adults. Self-rated religiosity and spirituality were assessed using separate measures (although the measure used to assess the latter is explicitly ‘theistic’). Nadal et al. (2018) constructed four R/S identity groups that correspond with MRTS, ERAS, MSTR, and NRNS, and found that the ERAS and SBNR groups reported higher psychological well-being than the other two groups. However, psychological well-being in the ERAS group was comparable to that of the SBNR group.

Illuminating the complex relationship between spirituality and psychological well-being, a recent longitudinal study of New Zealand adults (Highland et al., 2022) found that “belief in a spirit or life force predicts lower personal well-being and life satisfaction” during the first waves, but toward the end of the 10-year study period the study revealed that “belief (relative to disbelief) in a spirit or life force predicts increasing personal well-being and life satisfaction over time” (p. 1738). Thus, it is reasonable to conclude that SBNR and ERAS were most closely associated with an increase in psychological well-being, even though spirituality was assessed by specific beliefs (namely belief in a spirit or life force) rather than by R/S self-identification.

Other studies suggest the implications of self-identifying as SBNR for mental health and psychological well-being may be more negative. In a cross-sectional study with a national sample of United Kingdom adults, King et al. (2013) examined associations between a spiritual or religious understanding of life (i.e., religious, spiritual, neither) and a variety of psychiatric symptoms and diagnoses (e.g., generalized anxiety disorder). They reported evidence supporting some negative psychological outcomes associated with self-identifying exclusively as spiritual, suggesting that “people who profess spiritual beliefs in the absence of a religious framework are more vulnerable to mental disorder” (p. 72). Along similar lines, a recent two-wave longitudinal study of late adolescents and early adults in the United States found that those who consistently self-identified as SBNR over time tended to report worse depression (Upenieks and Ford-Robertson, 2022). The authors concluded that “consistently identifying as SBNR was associated with worse physical and mental health relative to youth that were consistently religious” (p. 4635). These findings resonate with an earlier longitudinal study that used data from a national sample of middle-aged United States adults to investigate the risk of depression among those who identified more with spirituality than religion (Vittengl, 2018). Although overall spirituality in conjunction with religiosity did not predict subsequent depression, Vittengl (2018, p. 386) found that “greater spirituality than religiosity significantly predicted subsequent increases in depressive symptoms and risk for major depressive disorder”.

On the whole, prior empirical research on the psychological well-being of people who identify with spirituality, and in particular those who self-identify as MSTR, has yielded inconclusive findings. Additional evidence is likely to help with constructing a clearer picture of the linkages between self-identifying as SBNR/MSTR and psychological well-being.

#### Mystical experiences and spirituality

In one of the first studies that examined relations of religion, spirituality, and mystical experiences, Zinnbauer et al.’s (1997) cross-sectional study of United States adults evidenced a positive correlation between mystical experiences (assessed principally as ego-loss and unity experiences) and self-rated spirituality, but mystical experiences were not correlated with self-rated religiousness. Several years later, Hood (2003) reported from a study with undergraduate students in the United States that higher levels of mystical experiences were expressed by individuals who identified as MSTR. More broadly, the groups which included spirituality as a prominent part of their R/S self-identity (i.e., MSTR and ERAS) scored higher on mystical experiences than those for whom spirituality was less prominent (i.e., MRTS and NRNS). This finding illustrates that mysticism is associated with spirituality both within and outside traditional religiousness. Willard and Norenzayan (2017) included a measure of mystical experience in their cross-sectional study that profiled United States adults who self-identified as SBNR. Their findings suggested that, in comparison to those who self-identify as religious, “the SBNR, and spirituality more generally, is more experiential, including greater mystical experiences and feelings of universal connectedness” (p. 143). Similar conclusions have been drawn in subsequent studies, including evidence that individuals who identify as SBNR tend to score “higher than both nonreligious and religious in belief in God as a mystical cosmic force” (Johnson et al., 2018, p. 133). Strong relations of mystical experiences with both spiritual self-identification and self-rated spirituality have been reported in research that has dedicated more specific attention to the association between mystical experiences and spirituality (e.g., Streib et al., 2021; Streib and Chen, 2021), suggesting that spirituality is an “experience-oriented approach to unite with some kind of transcendence” (Klein et al., 2016, p. 184).

Overall, previous (mostly cross-sectional) research supports a clear connection between mystical experiences and self-identified spirituality. Although existing empirical findings resonate with the notion that “spirituality involves experience(s) of connection, including mystical experiences” (Wixwat and Saucier, 2021, p. 121), further study is needed to test this assumption and develop a more robust body of causal evidence linking R/S identities that include self-identified spirituality with mystical experiences.

### The present studies

In the current set of studies, we provide further evidence on similarities and distinctions between four R/S identities that differentiate individuals based on whether they describe themselves as MRTS, MSTR, ERAS, or NRNS. Using cross-sectional data from adults in Germany and the United States, we compare the abovementioned R/S identities on sociodemographic characteristics and a variety of concurrently assessed criterion variables that are considered relevant for understanding R/S self-identification (Study 1). With much of the existing evidence along these lines based on cross-sectional data, the results of Study 1 can corroborate or challenge the findings of previous work. To strengthen causal inferences about the effects of different R/S self-identifications on individual characteristics that are comparable to the criterion variables included in Study 1, we also use longitudinal data from German and United States adults to explore the associations of these four R/S identities with subsequent outcomes (Study 2).

## Study 1

Using cross-sectional data from adults living in Germany and the United States, we explore differences between four R/S self-identities—MRTS, MSTR, ERAS, and NRNS—on an array of sociodemographic characteristics (i.e., age, gender, education, country of residence, religious affiliation) and salient criterion variables (i.e., Big Five personality traits, psychological well-being, generativity, mystical experiences, religious schemata, self-rated religiosity and spirituality). Based on results from prior research, we anticipated that the MSTR self-identification would be more strongly associated with openness to experience and mysticism than the other R/S identities. Although we expected to find some evidence of differences between the R/S identities on other criterion variables, we did not have clear *a priori* expectations about the pattern of differences that might emerge.

### Methods

#### Sample

The sample in this study included *n* = 3,491 individuals who participated at a single point in time before 2017.^1^ About half of the sample (49.53%) was from the United States, with the remainder from Germany.^2^ Their average age was 36.51 years (SD = 15.56), 58.06% were female, and 41.64% had completed undergraduate education or above. More than half of the participants identified as Christian (57.46%); the remainder identified with other religions (17.33%) or indicated that they were not religious (25.21%).

#### Measures

Details about the measures that were used can be found in Supplemental Text 1 and Supplemental Table S1. Descriptive statistics and estimated internal consistency for each measure are reported in Supplemental Table S2. Prior research has documented evidence supporting the valid interpretation of scores on each measure not only in both United States and German samples, but also in a wide spectrum of samples from non-Western cultures (for more information, see Supplemental Text 1).

##### R/S identity

We used a single item to assess R/S identification: “Mark the statement below that most identifies you.” Participants selected one of four response options: “I am neither religious nor spiritual,” “I am equally religious and spiritual,” “I am more religious than spiritual,” or “I am more spiritual than religious”.

##### Criterion variables

We assessed the Big Five personality traits (i.e., neuroticism, extraversion, openness to experience, agreeableness, conscientiousness), six indices of psychological well-being (i.e., autonomy, environmental mastery, positive relations with others, personal growth, purpose in life, self-acceptance), generativity, three dimensions of mystical experiences (i.e., introvertive, extrovertive, interpretative), three religious schemata (i.e., truth of texts & teachings, fairness, tolerance, & rational choice, xenosophia/inter-religious dialog) and self-rated religiosity and spirituality. All criterion variables were continuous. To facilitate comparison of results across criterion variables, scores for each variable were transformed to a five-point scale (range: 1–5).

#### Data analysis

All analyses were conducted in R 4.1.3. We explored differences in sociodemographic characteristics and criterion variables between the four R/S identity groups. Categorial variables were cross tabulated with the R/S identities, and we used Chi-square tests of independence to examine whether the distribution of participants for each categorical variable differed across the R/S identities. For continuous variables, we report means and standard deviations for each of the four R/S identity groups, and one-way ANOVAs were used to test for evidence of differences between the groups. Effect sizes are represented in the metric of η^2^.

### Results

Table 1 reports the descriptive statistics for categorical and continuous variables by R/S identity. We found differences in R/S identity based on age (*p* < 0.001), such that the mean age was lower in the ERAS and MSTR groups. There was also evidence suggesting that the distributions for gender, highest level of education, country of residence, and religious affiliation varied by R/S identity (*p*s ≤ 0.019), with the largest effect size found for religious affiliation. Compared to adherents of Christianity and other religious traditions, nonreligious people were more likely to be in the NRNS group and very unlikely to be in the ERAS or MRTS groups. However, the percentages of individuals who identified as MSTR was somewhat similar in the three religious affiliation groups.

**Table 1:** Distribution of participant characteristics by religious/spiritual identity in Study 1.

Characteristic	Religious/spiritual identity				Effect size
	NRNS (*n* = 727)	ERAS (*n* = 922)	MRTS (*n* = 509)	MSTR (*n* = 1,333)	
**Sociodemographics**					
Age (years), *M* (SD)	37.83 (14.77) ^1^	35.05 (16.53) ^2^	40.01 (17.48) ^1^	35.47 (14.20) ^2^	0.013
Gender (%)					0.044
Male	28.07	23.91	14.14	33.88	
Female	15.59	28.22	14.90	41.29	
Highest level of education (%)					0.006
Below university education	22.53	26.51	14.19	36.77	
Undergraduate education or above	18.43	26.27	15.13	40.17	
Country of residence (%)					0.168
Germany	30.25	18.33	20.89	30.53	
United States	11.22	34.64	8.16	45.98	
Religious affiliation (%)					0.284
Nonreligious	49.77	4.09	2.05	44.09	
Christian	12.41	34.55	19.54	33.50	
Other religion	6.61	31.90	16.36	45.12	
**Big Five personality traits**					
Neuroticism, *M* (SD)	2.70 (0.75) ^1^	2.68 (0.63) ^1^	2.67 (0.61) ^1^	2.69 (0.70) ^1^	0.000
Extroversion, *M* (SD)	3.22 (0.60) ^3^	3.49 (0.51) ^1^	3.38 (0.51) ^2^	3.42 (0.55) ^2^	0.022
Openness to experience, *M* (SD)	3.76 (0.57) ^1^	3.35 (0.54) ^2^	3.29 (0.47) ^2^	3.74 (0.56) ^1^	0.127
Agreeableness, *M* (SD)	3.54 (0.52) ^2^	3.73 (0.47) ^1^	3.70 (0.47) ^1^	3.70 (0.49) ^1^	0.014
Conscientiousness, *M* (SD)	3.55 (0.57) ^2^	3.71 (0.54) ^1^	3.71 (0.50) ^1^	3.61 (0.56) ^2^	0.011
**Psychological well-being**					
Autonomy, *M* (SD)	3.76 (0.58) ^1^	3.61 (0.56) ^2^	3.50 (0.54) ^3^	3.71 (0.58) ^1^	0.020
Environmental mastery, *M* (SD)	3.45 (0.70) ^2^	3.59 (0.59) ^1^	3.58 (0.60) ^1^	3.51 (0.63) ^12^	0.006
Personal growth, *M* (SD)	4.07 (0.55) ^2^	4.01 (0.52) ^2^	3.87 (0.48) ^3^	4.18 (0.53) ^1^	0.041
Positive relations with others, *M* (SD)	3.70 (0.67) ^3^	3.96 (0.58) ^1^	3.82 (0.58) ^2^	3.93 (0.61) ^1^	0.020
Purpose in life, *M* (SD)	3.67 (0.63) ^3^	3.87 (0.57) ^1^	3.83 (0.52) ^12^	3.78 (0.60) ^2^	0.012
Self-acceptance, *M* (SD)	3.58 (0.71) ^2^	3.73 (0.60) ^1^	3.63 (0.59) ^12^	3.73 (0.63) ^1^	0.008
Generativity, *M* (SD)	2.79 (0.46) ^2^	2.97 (0.41) ^1^	2.80 (0.38) ^2^	2.94 (0.43) ^1^	0.025
**Mystical experiences**					
Introvertive, *M* (SD)	2.55 (1.02) ^4^	3.60 (0.81) ^2^	3.07 (0.81) ^3^	3.76 (0.88) ^1^	0.203
Extrovertive, *M* (SD)	2.43 (1.03) ^4^	3.41 (0.95) ^2^	2.85 (0.92) ^3^	3.66 (0.99) ^1^	0.176
Interpretive, *M* (SD)	2.79 (0.83) ^3^	4.03 (0.67) ^1^	3.60 (0.72) ^2^	3.94 (0.75) ^1^	0.255
**Religious schemata**					
Truth of texts & teachings, *M* (SD)	1.94 (0.95) ^4^	3.56 (0.89) ^1^	3.38 (0.84) ^2^	2.56 (1.13) ^3^	0.277
Fairness, tolerance, & rational choice, *M* (*SD*)	3.99 (0.76) ^3^	4.11 (0.57) ^2^	4.11 (0.57) ^12^	4.20 (0.61) ^1^	0.015
Xenosophia/inter-religious dialog, *M* (SD)	3.00 (0.72) ^3^	3.38 (0.77) ^2^	3.30 (0.68) ^2^	3.62 (0.78) ^1^	0.090
Religiosity, *M* (SD)	1.29 (0.54) ^3^	4.06 (0.89) ^1^	3.89 (0.84) ^1^	2.09 (1.10) ^2^	0.594
Spirituality, *M* (SD)	1.41 (0.67) ^3^	4.22 (0.79) ^1^	2.51 (1.05) ^2^	4.27 (0.83) ^1^	0.706

NRNS = neither religious nor spiritual, ERAS = equally religious and spiritual, MRTS = more religious than spiritual, MSTR = more spiritual than religious. *n* = 3,491 for all analyses. For categorical variables, Chi-square tests of independence were used to examine the proportion of individuals in each R/S identity groups with that characteristic. For continuous variables, differences in means were tested using one-way ANOVA. Effect sizes are in the metric of η^2^. Post-hoc pairwise comparisons of group means were tested using Tukey HSD tests. Superscripts indicate both ranking and difference. Groups with dissimilar superscripts are significantly different at *p* < 0.05, and groups with the same or overlapping superscripts are not statistically different from one another.

When examining the criterion variables, we found evidence of differences between the R/S identities on the Big Five personality traits of extraversion, openness to experience, agreeableness, and conscientiousness (*p*s < 0.001) but not neuroticism (*p* = 0.945). The largest effect size emerged for openness to experience, which was also one of the largest effect sizes across the set of criterion variables we examined. Extraversion was highest in the ERAS group, the NRNS and MSTR groups scored highest on openness to experience, the ERAS, MRTS, and MSTR groups scored highest on agreeableness, and conscientiousness was highest in both the ERAS and MRTS groups.

We found some evidence of differences between the R/S identities on all six indices of psychological well-being (*p*s ≤ 0.002), although effect sizes were among the smallest of those observed for the criterion variables included in this study. The MRTS group had the lowest mean on the autonomy and personal growth subscales. The NRNS and MSTR groups scored lowest on environmental mastery, the NRNS and MRTS groups scored lowest on self-acceptance, and the NRNS group scored lowest on positive relations with others and purpose in life. Differences were also found on the generativity criterion variable (*p* < 0.001), with higher mean scores in the ERAS and MSTR groups.

Large differences were found between the R/S identities on the three mystical experiences scales (*p*s < 0.001), all of which were among the largest observed for any of the criterion variables we examined. The MSTR group scored highest on both introvertive and extrovertive mysticism, while the ERAS and MSTR groups scored highest on interpretive mysticism.

There were differences between the R/S identities on the three religious schema variables of truth of texts & teachings, fairness, tolerance, & rational choice, and xenosophia/inter-religious dialog (*p*s < 0.001). The largest effect size was found for truth of texts & teachings, which was also one of the largest effect sizes that emerged across the set of criterion variables we examined. The NRNS group scored lowest on each factor, although the mean score for truth of texts & teachings was also comparably low in the MSTR group. In contrast, the MSTR group scored highest on xenosophia/inter-religious dialog.

Self-rated religiosity and spirituality evidenced the largest effect sizes of any criterion variable (*p*s < 0.001), with the results providing evidence validating R/S identity. Religiosity was highest in the ERAS and MRTS groups, and spirituality was highest in the ERAS and MSTR groups. The NRNS group scored lowest on both self-rated religiosity and spirituality.

### Discussion

Consistent with our expectations, cross-sectional analyses in Study 1 provided evidence supporting some differences between the R/S identities of MRTS, MSTR, ERAS, and NRNS on various sociodemographic characteristics and criterion variables that were examined. We center our discussion on the findings for which there was especially strong evidence of differences between one or more R/S identities, including religious affiliation, self-rated religiosity and spirituality, the Big Five personality trait of openness to experience, the three dimensions of mystical experiences, and selected religious schemata.

Differences in the distribution of religious affiliation by R/S identity provide insight into the heterogeneity of religious affiliation among the MSTR group, with almost half (44.09%) of the ‘religious nones’ self-identifying as MSTR (approximately half of the ‘religious nones’ self-identified as NRNS). This finding indicates that, for many participants, the MSTR identity reflects distance or opposition to organized religion. However, approximately 33.50% of Christians and 45.12% of those who affiliated with other religious traditions self-identified as MSTR, indicating that many individuals who are MSTR do affiliate with a religion. Moreover, the mean for self-rated religiosity in this group was not the lowest of the four R/S identities and the variance of self-rated religiosity scores in the MSTR group was greater than each of the other three R/S identity categories. Taken together, these findings resonate with prior work that suggests the spirituality of those who identify as MSTR is heterogeneous (Wixwat and Saucier, 2021).

Interestingly, the pattern of findings that we observed for religious affiliations in this study roughly corresponds with qualitative research that has found people’s own definitions of spirituality generally fall into one of three broad categories: (a) a more ‘religious’ understanding that associates spirituality with God, Jesus, the Bible, system, and organization; and (b) an understanding of spirituality as connectedness with other humans, the inner or higher self, nature, and the universe; or (c) an understanding that relates spirituality to morality/ethics and an orientation toward humanity (e.g., Ammerman, 2013; Eisenmann et al., 2016; Steensland et al., 2018). Although the analyses in this study cannot one-to-one replicate the diverse descriptions of spirituality that have emerged via qualitative methods, our findings clearly document that the spirituality of those who identify as MSTR may or may not be co-present with religion (*cf.* Schnell, 2012).

We found that the mean for the Big Five personality trait of openness to experience was higher among the MSTR group than the MRTS and ERAS groups. This finding aligns with previous research that has documented evidence suggesting that openness to experience is positively associated with self-rated spirituality but is negatively associated with religiousness (Saroglou, 2002, 2010; Saucier and Skrzypińska, 2006; Saroglou and Muñoz-García, 2008; Schnell, 2012; Streib et al., 2016).

Some of the largest differences found between the R/S identities were on the dimensions of mystical experiences. The means for the introvertive and extrovertive mysticism subscales were highest among the MSTR group, which coincides with previous evidence indicating that mystical experiences tend to be more frequently endorsed by those who identify as MSTR (Hood, 2003). More generally, the pattern of findings that we observed supported differences in mystical experiences between the two groups for whom spirituality is a more prominent feature of their R/S self-identity (i.e., MSTR, ERAS) compared to the two groups for whom spirituality is less prominent (i.e., MRTS, NRNS). Our findings also resonate with other studies that have documented evidence of a strong correlation between spirituality and mysticism (Zinnbauer et al., 1997; Klein et al., 2016; Willard and Norenzayan, 2017; Streib et al., 2021; Streib and Chen, 2021; Upenieks and Ford-Robertson, 2022).

On the religious schemata dimensions, the MSTR group had the second lowest mean for truth of texts & teachings but the highest mean for xenosophia/inter-religious dialog. Thus, the spirituality of individuals who identify as MSTR might be considered anti-fundamentalist and open to dialog concerning religious matters. These findings provide a unique perspective on spirituality that has not been widely documented in empirical research, although a similar distinction between fundamentalist religiosity and ‘spirituality/mature faith’ was applied in Saroglou’s (2010) meta-analysis—which our findings seem to support.

Taken together, Study 1 provides additional evidence corroborating the findings of prior research and offers more novel insight into differences between R/S identities on key personal characteristics. Most notably, a considerable proportion of religiously affiliated and non-affiliated individuals identified as MSTR, and the MSTR group differed from the other three R/S identities on several criterion variables (e.g., introvertive mysticism, xenosophia/inter-religious dialog). However, the cross-sectional data precludes inferences of causality, which is an issue we seek to address (as far as is possible with observational data) in Study 2.

## Study 2

To date, only a few studies have reported evidence concerning the cross-time associations of R/S identities with subsequent outcomes, with most studies focusing on outcomes within the broad domain of psychological well-being (e.g., Highland et al., 2022; Upenieks and Ford-Robertson, 2022). In Study 2, we extended the cross-sectional findings of Study 1 to a longitudinal design estimating the effects of the four R/S identities on a wide range of subsequent outcomes across the Big Five personality traits, psychological well-being, generativity, mystical experiences, religious schemata, self-rated religiosity and spirituality, religious centrality, God representation, and cognitive style.

### Methods

#### Sample

The sample included *n* = 751 German (60.99%) and United States (39.01%) adults who participated longitudinally in the Bielefeld-Chattanooga Longitudinal Study of Faith Development. Their average age was 38.80 years (SD = 17.24), a majority of whom were female (56.72%) and had completed undergraduate education or above (64.85%). Approximately one third of participants indicated that they were not religiously affiliated (37.55%), with the remainder either identifying as Christian (43.94%) or as an adherent of another religion (18.51%).

#### Outcomes

All criterion variables in Study 1 were included as outcomes in Study 2, each of which was assessed in all three waves. Three additional outcome variables were measured in Waves 2 and 3: intolerance of ambiguity (Budner, 1962), need for cognition (Cacioppo et al., 1984), and religious centrality (Huber and Huber, 2012). God representation, referring to the perception of whether God is authoritarian, benevolent, mystical, and ineffable (Silverman et al., 2016; Johnson et al., 2018), was assessed as an outcome in Wave 3. Detailed descriptions of the measures used to assess the outcomes can be found in Supplemental Text 1, and further information about the timing of assessments is reported in Supplemental Table S1. Descriptive statistics and estimated internal consistency for the outcomes are presented in Supplemental Table S2.

#### Data analysis

We employed the outcome-wide longitudinal design, which is a rigorous analytic approach for estimating potential causal effects of a single exposure on a wide range of subsequent outcomes (VanderWeele et al., 2020). In a series of multiple linear regression analyses, we regressed continuous scores of each outcome (27 outcomes in total) assessed in Wave 3 on R/S identity assessed in Wave 2 (one outcome at a time). The average length of time between Wave 2 and Wave 3 was 2.89 years. Each model controlled for prior values of all available outcomes (i.e., Big Five personality traits, indices of psychological well-being, generativity, dimensions of mysticism, religious schemata, self-ratings of religiosity and spirituality) assessed in Wave 1. The average length of time between Wave 1 and Wave 2 was 5.63 years. All models also adjusted for sociodemographic characteristics of gender (female vs. male), country of residence (United States vs. Germany), highest level of education (below university education vs. undergraduate education or above), and religious affiliation (Christian vs. other religion vs. nonreligious) assessed in Wave 2.

All analyses were conducted using the *lavaan* package in R 4.1.3. We used a full-information maximum likelihood estimator to compute parameter estimates (Enders, 2010). All outcomes were standardized (mean = 0, standard deviation = 1) to allow for effect sizes to be compared across outcomes. The multinomial R/S identity variable was contrast coded into dummy variables, such that regression coefficients for each outcome estimate the mean difference in the standardized scores of an outcome between the specific R/S identity group and the grand mean.

### Results

Effect estimates for associations of the R/S identities with each of the subsequent outcomes are reported in Table 2. After adjusting for multiple testing, some robust associations were found for introvertive and extrovertive mysticism, self-rated religiosity and spirituality, religious centrality, and authoritarian God representation. In particular, the MSTR group scored higher on subsequent introvertive mysticism (*β* = 0.32, 95% CI = 0.12, 0.51), extrovertive mysticism (*β* = 0.32, 95% CI = 0.14, 0.51), and spirituality (*β* = 0.55, 95% CI = 0.38, 0.71) compared to the grand mean, but scored lower on subsequent religiosity (*β* = −0.32, 95% CI = −0.49, −0.15). The NRNS group scored lower on subsequent religiosity (*β* = −0.46, 95% CI = −0.68, −0.24), spirituality (*β* = −0.37, 95% CI = −0.60, −0.15), and religious centrality (*β* = −0.50, 95% CI = −0.71, −0.28), as well as higher on subsequent authoritarian God representation (*β* = 0.55, 95% CI = 0.27, 0.83), compared to the grand mean. In addition, the MRTS group scored higher on subsequent religiosity (*β* = 0.50, 95% CI = 0.27, 0.73) and lower on subsequent spirituality (*β* = −0.41, 95% CI = −0.63, −0.19) relative to the grand mean.

**Table 2:** Associations of religious/spiritual identity (Wave 2) with subsequent outcomes (Wave 3) in Study 2.

Outcome	Religious/spiritual identity			
	NRNS (*n* = 232) *β* [95% CI]	ERAS (*n* = 163) *β* [95% CI]	MRTS (*n* = 69) *β* [95% CI]	MSTR (*n* = 287) *β* [95% CI]
**Big Five personality traits**				
Neuroticism	−0.06 [−0.32, 0.21]	0.10 [−0.12, 0.33]	−0.08 [−0.36, 0.19]	0.04 [−0.17, 0.24]
Extroversion	−0.11 [−0.33, 0.11]	−0.05 [−0.23, 0.12]	0.07 [−0.14, 0.29]	0.09 [−0.08, 0.25]
Openness to experience	−0.01 [−0.26, 0.23]	−0.09 [−0.29, 0.12]	0.03 [−0.22, 0.28]	0.07 [−0.12, 0.27]
Agreeableness	−0.03 [−0.30, 0.24]	−0.02 [−0.24, 0.20]	−0.25 [−0.52, 0.01]	0.30^*^ [0.11, 0.49]
Conscientiousness	0.19 [−0.04, 0.42]	−0.25^*^ [−0.44, −0.07]	−0.14 [−0.37, 0.09]	0.20^*^ [0.03, 0.38]
**Psychological well-being**				
Autonomy	0.02 [−0.22, 0.26]	0.02 [−0.18, 0.22]	0.15 [−0.11, 0.40]	−0.18 [−0.36, 0.00]
Environmental mastery	0.01 [−0.29, 0.31]	−0.17 [−0.41, 0.07]	0.28 [−0.02, 0.59]	−0.12 [−0.35, 0.10]
Personal growth	0.05 [−0.22, 0.33]	−0.10 [−0.33, 0.13]	−0.02 [−0.31, 0.26]	0.07 [−0.14, 0.28]
Positive relations with others	0.20 [−0.03, 0.43]	−0.11 [−0.30, 0.09]	0.00 [−0.24, 0.25]	−0.09 [−0.28, 0.09]
Purpose in life	−0.12 [−0.38, 0.14]	−0.09 [−0.30, 0.12]	0.07 [−0.19, 0.34]	0.14 [−0.06, 0.33]
Self-acceptance	−0.02 [−0.31, 0.27]	−0.16 [−0.39, 0.07]	0.19 [−0.11, 0.49]	−0.01 [−0.23, 0.21]
Generativity	−0.05 [−0.30, 0.21]	−0.12 [−0.33, 0.10]	−0.02 [−0.28, 0.23]	0.19 [−0.00, 0.38]
**Mystical experiences**				
Introvertive	−0.08 [−0.34, 0.17]	0.06 [−0.15, 0.28]	−0.30^*^ [−0.56, −0.04]	0.32^**^ [0.12, 0.51]
Extrovertive	−0.10 [−0.35, 0.15]	0.03 [−0.17, 0.24]	−0.26^*^ [−0.51, −0.02]	0.32^**^ [0.14, 0.51]
Interpretive	−0.27^*^ [−0.51, −0.03]	0.18 [−0.01, 0.37]	−0.15 [−0.38, 0.08]	0.25^*^ [0.07, 0.42]
**Religious schemata**				
Truth of texts & teachings	−0.26^*^ [−0.50, −0.02]	0.02 [−0.17, 0.22]	0.20 [−0.02, 0.43]	0.04 [−0.13, 0.21]
Fairness, tolerance, & rational choice	−0.03 [−0.32, 0.26]	0.02 [−0.23, 0.28]	−0.05 [−0.35, 0.24]	0.07 [−0.16, 0.29]
Xenosophia/inter-religious dialog	0.01 [−0.23, 0.24]	0.08 [−0.12, 0.28]	−0.27^*^ [−0.49, −0.04]	0.18 [−0.00, 0.35]
Religiosity	−0.46^**^ [−0.68, −0.24]	0.28^*^ [0.09, 0.47]	0.50^**^ [0.27, 0.73]	−0.32^**^ [−0.49, −0.15]
Spirituality	−0.37^**^ [−0.60, −0.15]	0.24^*^ [0.05, 0.42]	−0.41^**^ [−0.63, −0.19]	0.55^**^ [0.38, 0.71]
Religious centrality^#^	−0.50^**^ [−0.71, −0.28]	0.12 [−0.05, 0.29]	0.18 [−0.03, 0.39]	0.20^*^ [0.04, 0.35]
**God representation^#^**				
Authoritarian	0.55^**^ [0.27, 0.83]	−0.18 [−0.42, 0.07]	−0.18 [−0.48, 0.13]	−0.19 [−0.42, 0.03]
Benevolent	−0.22 [−0.49, 0.05]	0.11 [−0.12, 0.34]	0.07 [−0.22, 0.36]	0.04 [−0.17, 0.25]
Mystical	−0.10 [−0.38, 0.19]	−0.06 [−0.30, 0.18]	−0.18 [−0.47, 0.12]	0.33^*^ [0.11, 0.54]
Ineffable	0.34^*^ [0.01, 0.66]	0.09 [−0.18, 0.37]	−0.34^*^ [−0.68, −0.00]	−0.09 [−0.34, 0.16]
**Cognition^#^**				
Intolerance of ambiguity	−0.28 [−0.56, 0.01]	0.28^*^ [0.03, 0.52]	0.02 [−0.28, 0.32]	−0.02 [−0.24, 0.21]
Need for cognition	0.21 [−0.07, 0.49]	−0.27^*^ [−0.50, −0.04]	0.04 [−0.25, 0.33]	0.02 [−0.19, 0.24]

NRNS = neither religious nor spiritual, ERAS = equally religious and spiritual, MRTS = more religious than spiritual, MSTR = more spiritual than religious, β = standardized difference from the grand mean, CI = confidence interval. *n* = 751 for all analyses. In separate models, each Wave 3 outcome was regressed on religious/spiritual identity reported in Wave 2. Regression models estimated the mean difference in the standardized scores of each outcome between the specific religious/spiritual identity group and the grand mean. All models adjusted for gender (female vs. male), country of residence (United States vs. Germany), highest level of education (below university education vs. undergraduate education or above), and religious affiliation (Christian vs. other religion vs. nonreligious) assessed in Wave 2, and prior values of all available outcome variables assessed in Wave 1 (^#^religious centrality, God representation, and cognition variables were not measured in Wave 1). ^*^p < 0.05 before Bonferroni correction, ^**^p < 0.05 after Bonferroni correction (the *p*-value cutoff for Bonferroni correction was 0.05/27 = 0.0019 for each outcome).

Evidence of other effects also emerged, but associations were more modest. Compared to the grand mean, the NRNS group scored lower on subsequent interpretive mysticism (*β* = −0.27, 95% CI = −0.51, −0.03) and truth of texts & teachings (*β* = −0.26, 95% CI = −0.50, −0.02), but scored higher on subsequent ineffable God representation (*β* = 0.34, 95% CI = 0.01, 0.66). The ERAS group scored lower on subsequent conscientiousness (*β* = −0.25, 95% CI = −0.44, −0.07) and need for cognition (*β* = −0.27, 95% CI = −0.50, −0.04) compared to the grand mean, but scored higher on subsequent religiosity (*β* = 0.28, 95% CI = 0.09, 0.47), spirituality (*β* = 0.24, 95% CI = 0.05, 0.42), and intolerance of ambiguity (*β* = 0.28, 95% CI = 0.03, 0.52). The MRTS group scored lower on subsequent introvertive mysticism (*β* = −0.30, 95% CI = −0.56, −0.04), extrovertive mysticism (*β* = −0.26, 95% CI = −0.51, −0.02), xenosophia/inter-religious dialog (*β* = −0.27, 95% CI = −0.49, −0.04), and ineffable God representation (*β* = −0.34, 95% CI = −0.68, −0.00) relative to the grand mean. The MSTR group scored higher on subsequent agreeableness (*β* = 0.30, 95% CI = 0.11, 0.49), conscientiousness (*β* = 0.20, 95% CI = 0.03, 0.38), interpretive mysticism (*β* = 0.25, 95% CI = 0.07, 0.42), religious centrality (*β* = 0.20, 95% CI = 0.04, 0.35), and mystical God representation (*β* = 0.33, 95% CI = 0.11, 0.54) compared to the grand mean. Other differences between subsequent mean scores for the R/S identities and the grand mean on the outcomes were more negligible, although confidence intervals tended to relatively wide and included a range of meaningful values that could reasonably contain true effect estimates.

### Discussion

In our longitudinal analysis estimating associations of the four R/S identities with numerous subsequent outcomes, the findings of Study 2 provided further evidence supporting differences between the self-identities of MRTS, MSTR, ERAS, and NRNS. We center the discussion on some key findings that we observed for the MSTR group, as this R/S self-identity is of primary interest in the present investigation.

At first glance, the association that we observed for the MSTR self-identity in Wave 2 with subsequent religiosity in Wave 3 appears contradictory. On the one hand, the association between the MSTR self-identity and self-rated religiosity was negative. Together with the positive association between the MSTR self-identity and self-rated spirituality, this may indicate that the change toward becoming ‘more spiritual’ and ‘less religious’ could be relatively stable, or perhaps increasing, over the three-year follow-up period. On the other hand, the association of the MSTR self-identity in Wave 2 with centrality of religiosity in Wave 3 was positive. This apparent contradiction might be explained by the items that were used to measure religious centrality. Specifically, two pairs of items, namely two items for religious experience and two items for private religious practice, assess either a theistic or inter-religious version of religiosity. For each pair of items, the item that receives the highest endorsement is included in the total score of religious centrality. Thus, rather than signaling a contradiction, our findings for the self-rated religiosity and religious centrality of the MSTR group suggests that identifying as MSTR might not engender opposition to religiosity, if it includes the inter-religious dimension. This notion is consistent with the assumption that spirituality can include vertical and horizontal transcendence (Schnell, 2012; Mercadante, 2014; Streib and Hood, 2016b). Based on the findings of Study 2, it is possible that individuals who self-identify as MSTR tend to develop a stable (or perhaps even increasing) experiential and privately practiced religiosity, which can be vertical or horizontal (or both).

## General discussion

Consistent with previous research (e.g., Saroglou, 2010; Streib et al., 2016), the findings of Study 1 indicated that the Big Five personality trait of openness to experience tended to be higher among individuals who self-identify as MSTR and lower among those who identify as MRTS or ERAS. However, we found little evidence to suggest that any of the R/S identities in Wave 2 were associated with change in openness to experience by Wave 3. One potential reason for these contrasting findings is that there may have been relatively little change in openness to experience from Wave 1 to Wave 3, which aligns with research that has shown openness to experience tends to be relatively stable over time during adulthood (McCrae et al., 2021). Although the findings of Study 2 suggest that changes in openness to experience may not be predicted by R/S identity during adulthood, the strong correlations documented in Study 1 do not preclude the possibility that openness to experience may predict subsequent preferences for spirituality (see Wink et al., 2007). Given that our Study 2 findings are limited to R/S identities reported at a single point in time, additional research is needed to determine whether our findings replicate in other samples and stages of the life course.

For the MSTR group, we did find somewhat similar evidence in Studies 1 and 2 concerning the Big Five personality traits of conscientiousness and agreeableness. Specifically, the mean scores on these two personality traits were among the highest of any R/S identity group for those who identified as MSTR, and the MSTR group scored higher on subsequent conscientiousness and agreeableness in Study 2. These findings could suggest that people who identify as MSTR are not only relativistic and explorative, as their high scores on openness to experience may suggest, but their worldview may consolidate over time and engender higher agreeableness and conscientiousness. This interpretation echoes the Study 2 finding of an increase in religious centrality among those who identified as MSTR. However, proposing that a ‘more spiritual’ self-understanding might develop into a commitment to spiritual praxis and a more solidified worldview is admittedly speculative and in need of further empirical investigation.

One of the strongest correlates (Study 1) and outcomes (Study 2) associated with self-identifying as MSTR was mysticism. This evidence was consistent across all three dimensions of mystical experiences, with particularly robust findings emerging in Study 2 for introvertive and extrovertive mysticism. In contrast, the findings of Study 2 indicated that the MRTS self-identity was negatively associated with change in introvertive and extrovertive mysticism, and the NRNS self-identity was negatively associated with interpretative mysticism. Taken together, the findings of both studies align closely with previous research (Zinnbauer et al., 1997; Hood, 2003; Klein et al., 2016; Willard and Norenzayan, 2017; Streib et al., 2021; Streib and Chen, 2021; Upenieks and Ford-Robertson, 2022), but add useful information about the potential longitudinal effects of the MSTR identity. Similar to our findings for the associations of the MSTR identity with dimensions of mystical experiences, Study 2 also documented evidence indicating that the MSTR self-identity was associated with a subsequent increase in representing God as mystical. This finding resonates with research by Johnson et al. (2018), who reported that “SBNRs were significantly higher than both nonreligious and religious in belief in God as a mystical cosmic force” (p. 133).

Although means for the indices of psychological well-being in the MSTR group in Study 1 were consistently among the highest, the only psychological well-being criterion on which individuals who identified as MSTR scored higher than the other R/S identities was personal growth. In Study 2, we found little evidence to suggest that the R/S identities were associated with subsequent psychological well-being outcomes. These findings generally diverge from previous studies that have either documented a positive (Nadal et al., 2018; Highland et al., 2022) or a negative (King et al., 2013; Vittengl, 2018; Upenieks and Ford-Robertson, 2022) association of spirituality or spiritual self-identification with indicators of psychological well-being. Evidence of null, positive, or negative associations could be due to differences in methodology (e.g., samples, measures, analytic approaches) across studies. For example, some studies have used mental health diagnoses derived from clinical interviews (e.g., King et al., 2013), whereas others have used single-item self-report measures to assess psychological outcomes (e.g., Upenieks and Ford-Robertson, 2022). Recent evidence suggests that the lag between assessment of R/S identity and psychological well-being outcomes might be particularly important for observing associations longitudinally. Specifically, Highland et al.’s (2022) longitudinal analysis with data spanning about a decade indicated that personal well-being and life satisfaction tends to increase at a faster rate over time among those with a ‘more spiritual’ identity compared to non-believers. Extrapolating from these findings, it is possible that our lag of approximately three years between assessments of R/S identity and the outcomes may not be sufficient to observe associations that are more similar to the positive longer-term implications of a predominantly ‘more spiritual’ R/S identity reported by Highland et al. (2022). However, additional study is needed to explore these speculative possibilities further.

### Limitations

This set of studies is not without limitations. Both studies had sufficient coverage of certain sociodemographic characteristics (e.g., age groups and gender), but the samples were not representative of general populations in the United States and Germany. Therefore, the generalizability of our findings may be limited. In addition, our studies included participants entirely from Western, Educated, Industrialized, Rich, and Democratic (WEIRD) contexts, and it is unclear whether our findings are transportable to people living in less WEIRD societies (Wong and Cowden, in press). This limitation is shared by much of the previous empirical literature on religion and spirituality more broadly (Captari et al., 2022; Counted et al., 2022; Cowden et al., 2023), but also with research on R/S self-identification and SBNR more specifically. We concur with Wixwat and Saucier’s (2021) insight that “understandings of what it means to be ‘spiritual but not religious’ should be attentive to potential cultural bias. The ‘modern spirituality’ … distinct from conventional religiousness that manifests in Western populations may be a poor representation of spirituality outside of Western culture” (p. 123). Indeed, the concepts of religion and spirituality may well have different meanings in non-Western religions. For instance, Muslim spirituality does not imply that spirituality exists separate from religion, as is common in the West. The Western version of ‘spirituality’ is translated in Iran as *manawiat*, referring to a search for a hidden, nonmaterialistic meaning that exists within the ultimate implications of human intentions and actions. Despite the different meanings that *manawiat* may have carried, empirical research has shown that people who identify with both religiousness and *manawiat* tend to report higher levels of psychological functioning than those who identify with only one of the two (Ghorbani et al., 2019). Along similar lines, a recent longitudinal study of Pakistanis during Ramadan showed that religiousness and spirituality mutually influenced each other over time (Chen et al., 2022). These findings highlight the importance of studying religion and spirituality in non-Western populations, which will likely require making use of research designs and measures that are sensitive to culture and context.

Our findings from Study 1 are based on cross-sectional data and cannot be used to infer causality. We attempted to address this concern in Study 2 using longitudinal data, and applied a rigorous analytic template to estimate associations of the four R/S identities in Wave 2 with Wave 3 outcomes that were included in Study 1 as criterion variables. By adjusting for Wave 1 values of these outcomes in Study 2, we were able to estimate the effects of the R/S identities on *change* in those outcomes. However, we included several additional Wave 3 outcomes in Study 2 (i.e., centrality of religiosity, God representation, intolerance of ambiguity, need for cognition) that were not assessed in Wave 1, increasing the risk of reverse causation for those outcomes. We were also limited in our ability to adjust for covariates that could reasonably confound the effect estimates observed for one or more outcomes (e.g., public religious participation), which ought to be considered when interpreting our findings.

## Conclusion

The present set of studies contribute to improving our understanding of the differences between individuals who identify as MSTR and the three other broad categories of R/S self-identifications that we examined (i.e., MRTS, ERAS, NRNS). Our findings point to the MSTR self-identity as a heterogenous group comprising those who may or may not affiliate with a particular religious tradition. What then are the common characteristics that support the coherence of the MSTR category of R/S self-identification?

Based on the findings of our studies, there appear to be several factors that unify the MSTR self-identity, including high trait of openness to experience, a readiness to engage in inter-religious dialog, and rejection of a highly structured version of religion in favor of a more ‘universalist’ version. Individuals who identify as MSTR tend to emphasize the experiential dimension of spirituality, as evidenced by the consistently strong associations we observed with mysticism and comparably high endorsement of a mystical image of God. The latter finding resonates with vertical and horizontal transcendence, such that a mystical God representation extends beyond the transcendence of supernatural beings to include targets within this world, such as nature or the universe, which is consistent with the experiential dimension reflected in extrovertive mysticism.

With previous research suggesting that mysticism is predictive of self-rated spirituality (Klein et al., 2016; Streib et al., 2021), and some evidence supporting mysticism as both a moderator and mediator of the association between self-rated religiosity and spirituality (Streib and Chen, 2021), our findings offer additional insight into potential effects of a ‘more spiritual’ orientation on mystical experiences over time. Collectively, such evidence points to a mutually reinforcing relationship between spirituality and mysticism, but further research is needed to test this theorizing and expand on the mechanisms by which they might be causally related.

## Data availability statement

The raw data for the original contributions presented in the study are publicly available. This data can be found at osf.io/afwux.

## Ethics statement

The research reported in these two studies was reviewed and approved by IRB University of Tennessee at Chattanooga Ethics Committee of Bielefeld University. The participants provided their written informed consent to participate in the research.

## Author contributions

HS and ZC contributed to conception and design of the study. HS organized the database and wrote the first draft of the manuscript. ZC performed the statistical analysis. RC and ZC wrote sections of the manuscript. All authors contributed to manuscript revision, read, and approved the submitted version.

## Funding

This publication was made possible through the support of grant 61834 from the John Templeton Foundation. The opinions expressed in this publication are those of the authors and do not necessarily reflect the views of the John Templeton Foundation. Data collection was supported by earlier grants from the German Research Foundation/Deutsche Forschungsgemeinschaft (DFG) and the John Templeton Foundation. Also, we acknowledge support for the publication costs by the Open Access Publication Fund of Bielefeld University and the DFG.

## Conflict of interest

The authors declare that the research was conducted in the absence of any commercial or financial relationships that could be construed as a potential conflict of interest.

## Publisher’s note

All claims expressed in this article are solely those of the authors and do not necessarily represent those of their affiliated organizations, or those of the publisher, the editors and the reviewers. Any product that may be evaluated in this article, or claim that may be made by its manufacturer, is not guaranteed or endorsed by the publisher.

## Supplementary material

The Supplementary material for this article can be found online at: https://www.frontiersin.org/articles/10.3389/fpsyg.2022.1025938/full#supplementary-material

## Footnotes

^1^Participants who completed a follow-up survey for the Bielefeld-Chattanooga Longitudinal Study of Faith Development were only included in Study 2.

^2^The selection of the United States and Germany as target populations can be explained historically. Specifically, this line of research was initiated in Germany at the turn of the century, with a focus on high-tension groups and their spirituality. This was the first study in Germany that assessed spirituality in a survey. At that time, the ability to compare German and United States samples was a step forward because many Germans considered spirituality an ‘American disease.’ Data collection efforts continued over time, yielding longitudinal data spanning approximately two decades.

## References

[ref1] Ammerman N. T. (2013). Spiritual but not religious? Beyond binary choices in the study of religion. J. Sci. Study Relig. 52, 258–278. doi: 10.1111/jssr.12024

[ref2] Berghuijs J. Pieper J. Bakker C. (2013). Conceptions of spirituality among the Dutch population. Arch. Psychol. Relig. 35, 369–397. doi: 10.1163/15736121-12341272

[ref3] Budner S. (1962). Intolerance of ambiguity as a personality variable. J. Pers. 30, 29–50. doi: 10.1111/j.1467-6494.1962.tb02303.x 13874381

[ref4] Cacioppo J. T. Petty R. E. Kao C. F. (1984). The efficient asessment of need for cognition. J. Pers. Assess. 48, 306–307. doi: 10.1207/s15327752jpa4803_13 16367530

[ref5] Captari L. E. Cowden R. G. Sandage S. J. Davis E. B. Bechara A. O. Joynt S.. (2022). Religious/spiritual struggles and depression during COVID-19 pandemic lockdowns in the global south: evidence of moderation by positive religious coping and hope. Psychol. Relig. Spiritual. 14, 325–337. doi: 10.1037/rel0000474

[ref6] Chen Z. J. Khan Z. Cowden R. G. Palitsky R. Huang Y. (2022). Call and response: a six-wave study of bidirectional links between religiosity and spirituality among Pakistani Muslims during Ramadan. Psychol. Relig. Spiritual. doi: 10.1037/rel0000479

[ref8] Counted V. Pargament K. I. Bechara A. O. Joynt S. Cowden R. G. (2022). Hope and well-being in vulnerable contexts during the COVID-19 pandemic: does religious coping matter? J. Posit. Psychol. 17, 70–81. doi: 10.1080/17439760.2020.1832247

[ref9] Cowden R. G. Counted V. Ho M. Y. (2023). “Positive psychology and religion/spirituality across cultures in Africa, Asia, and Oceania” in Handbook of Positive Psychology, Religion, and Spirituality. eds. Davis E. B. Worthington E. L. Jr. Schnitker S. A. (Cham, Switzerland: Springer), 243–259.

[ref10] Demmrich S. Huber S. (2020). What do seculars understand as ‘spiritual’? A replication of Eisenmann et al.’s semantics of spirituality. J. Relig. Eur. 13, 67–95. doi: 10.1163/18748929-13010008

[ref11] Eisenmann C. Klein C. Swhajor-Biesemann A. Drexelius U. Streib H. Keller B. (2016). “Dimensions of “spirituality”: the semantics of subjective definitions” in Semantics and Psychology of Spirituality: A Cross-cultural Analysis. eds. Streib H. R. W. Hood Jr. (Cham, Switzerland: Springer), 125–151.

[ref12] Enders C. K. (2010). Applied Missing Data Analysis. New York, NY: Guilford Press.

[ref13] Ghorbani N. Chen Z. J. Rabiee F. Watson P. J. (2019). Religious fundamentalism in Iran: religious and psychological adjustment within a Muslim cultural context. Arch. Psychol. Relig. 41, 73–88. doi: 10.1177/0084672419878832

[ref14] Harris K. A. Howell D. S. Spurgeon D. W. (2018). Faith concepts in psychology: three 30-year definitional content analyses. Psychol. Relig. Spiritual. 10, 1–29. doi: 10.1037/rel0000134

[ref15] Highland B. Worthington E. L. Jr. Davis D. E. Sibley C. G. Bulbulia J. A. (2022). National longitudinal evidence for growth in subjective well-being from spiritual beliefs. J. Health Psychol. 27, 1738–1752. doi: 10.1177/13591053211009280, PMID: 33855887

[ref17] Hood R. W. Jr. (2003). “The relationship between religion and spirituality” in Defining Religion: Investigating the Boundaries Between the Sacred and the Secular. eds. Greil A. L. Bromley D. G. (Amsterdam: Elsevier Science), 241–265.

[ref18] Houtman D. Aupers S. (2007). The spiritual turn and the decline of tradition: the spread of post-Christian spirituality in 14 western countries, 1981-2000. J. Sci. Study Relig. 46, 305–320. doi: 10.1111/j.1468-5906.2007.00360.x

[ref19] Houtman D. Tromp P. (2020). “Post-Christian spirituality: misconceptions, obstacles, prospects” in Assessing Spirituality and Religion in a Diversified World: Beyond the Mainstream Perspective. eds. Ai A. L. Harris K. A. Wink P. (Cham, Switzerland: Springer), 35–57.

[ref20] Huber S. Huber O. W. (2012). The centrality of the religiosity scale (CRS). Religion 3, 710–724. doi: 10.3390/rel3030710

[ref21] Hyman C. Handal P. J. (2006). Definitions and evaluation of religion and spirituality items by religious professionals: a pilot study. J. Relig. Health 45, 264–282. doi: 10.1007/s10943-006-9015-z

[ref22] Johnson K. A. Sharp C. A. Okun M. A. Shariff A. F. Cohen A. B. (2018). SBNR identity: the role of impersonal god representations, individualistic spirituality, and dissimilarity with religious groups. Int. J. Psychol. Relig. 28, 121–140. doi: 10.1080/10508619.2018.1416251

[ref23] King M. Marston L. McManus S. Brugha T. Meltzer H. Bebbington P. (2013). Religion, spirituality and mental health: results from a national study of English households. Br. J. Psychiatry 202, 68–73. doi: 10.1192/bjp.bp.112.112003, PMID: 23174516

[ref24] Klein C. Silver C. F. Coleman T. J. Streib H. Hood R. W. Jr. (2016). “"spirituality" and mysticism” in Semantics and Psychology of Spirituality: A Cross-cultural Analysis. eds. Streib H. Hood R. W. (Cham, Switzerland: Springer), 165–187.

[ref25] la Cour P. Ausker N. H. Hvidt N. C. (2012). Six understandings of the word spirituality in a secular country. Arch. Psychol. Relig. 34, 63–81. doi: 10.1163/157361212X649634

[ref26] Lindeman M. van Elk M. Lipsanen J. Marin P. Schjoedt U. (2019). Religious unbelief in three western European countries: identifying and characterizing unbeliever types using latent class analysis. Int. J. Psychol. Relig. 29, 184–203. doi: 10.1080/10508619.2019.1591140

[ref27] MacDonald D. A. (2000). Spirituality: description, measurement, and relation to the five factor model of personality. J. Pers. 68, 153–197. doi: 10.1111/1467-6494.t01-1-00094, PMID: 10820684

[ref28] McCrae R. R. De Bolle M. Löckenhoff C. E. Terracciano A. (2021). “Lifespan trait development: toward an adequate theory of personality” in The Handbook of Personality Dynamics and Processes. ed. Rauthmann J. F. (Cambridge, MA: Academic Press), 621–641.

[ref29] Mercadante L. (2014). Belief Without Borders: Inside the Minds of the Spiritual But Not Religious. Oxford, United Kingdom: Oxford University Press.

[ref30] Nadal A. R. C. Hardy S. A. Barry C. M. (2018). Understanding the roles of religiosity and spirituality in emerging adults in the United States. Psychol. Relig. Spiritual. 10, 30–43. doi: 10.1037/rel0000104

[ref31] Oman D. (2013). “Defining religion and spirituality” in Handbook of the Psychology of Religion and Spirituality. eds. Paloutzian R. F. Park C. L.. 2nd ed (New York, NY: The Guilford Press), 23–47.

[ref32] Pargament K. I. (1997). The Psychology of Religion and Coping: Theory, Research, Practice. New York, NY: The Guilford Press.

[ref33] Pargament K. I. Mahoney A. (2017). “Spirituality: the search for the sacred” in The Oxford Handbook of Positive Psychology. 3rd ed. eds. Snyder C. R. Lopez S. J. Edwards L. M. Marques S. C.. (Oxford, United Kingdom: Oxford University Press), 878–891.

[ref34] Saroglou V. (2002). Religion and the five factors of personality: a meta-analytic review. Personal. Individ. Differ. 32, 15–25. doi: 10.1016/S0191-8869(00)00233-6

[ref35] Saroglou V. (2010). Religiousness as a cultural adaptation of basic traits: a five-factor model perspective. Personal. Soc. Psychol. Rev. 14, 108–125. doi: 10.1177/1088868309352322, PMID: 20023209

[ref36] Saroglou V. Muñoz-García A. (2008). Individual differences in religion and spirituality: an issue of personality traits and/or values. J. Sci. Study Relig. 47, 83–101. doi: 10.1111/j.1468-5906.2008.00393.x

[ref37] Saucier G. Skrzypińska K. (2006). Spiritual but not religious? Evidence for two independent dispositions. J. Pers. 74, 1257–1292. doi: 10.1111/j.1467-6494.2006.00409.x, PMID: 16958702

[ref38] Schlehofer M. M. Omoto A. M. Adelman J. R. (2008). How do “religion” and “spirituality” differ? Lay definitions among older adults. J. Sci. Study Relig. 47, 411–425. doi: 10.1111/j.1468-5906.2008.00418.x

[ref39] Schnell T. (2012). Spirituality with and without religion. Differential relationships with personality. Arch. Psychol. Relig. 34, 33–61. doi: 10.1163/157361212X644495

[ref40] Silverman G. S. Johnson K. A. Cohen A. B. (2016). To believe or not to believe, that is not the question: the complexity of Jewish beliefs about god. Psychol. Relig. Spiritual. 8, 119–130. doi: 10.1037/rel0000065

[ref41] Simmons J. A. (2021). Religious, but not spiritual: a constructive proposal. Religion 12:433. doi: 10.3390/rel12060433

[ref42] Steensland B. Wang X. Schmidt L. C. (2018). Spirituality: what does it mean and to whom? J. Sci. Study Relig. 57, 450–472. doi: 10.1111/jssr.12534

[ref43] Streib H. Chen Z. J. (2021). Evidence for the brief mysticism scale: psychometric properties, and moderation and mediation effects in predicting spiritual self-identification. Int. J. Psychol. Relig. 31, 165–175. doi: 10.1080/10508619.2021.1899641

[ref45] Streib H. Hood R. W. Jr. (Eds.). (2016a). Semantics and Psychology of Spirituality. A Cross-cultural Analysis. Cham, Switzerland: Springer.

[ref44] Streib H. Hood R. W. Jr. (2016b). “Understanding “spirituality”—conceptual considerations” in Semantics and Psychology of Spirituality: A Cross-cultural Analysis. eds. Streib H. R. W. Hood Jr. (Cham, Switzerland: Springer), 3–17.

[ref47] Streib H. Hood R. W. Jr. Keller B. Csöff R.-M. Silver C. (2009). Deconversion. Qualitative and quantitative results from cross-cultural research in Germany and the United States of America. Göttingen: Vandenhoeck & Ruprecht.

[ref48] Streib H. Keller B. Bullik R. Steppacher A. Silver C. F. Durham M.. (2022). Deconversion Revisited. Biographical Studies and Psychometric Analyses Ten Years Later. Göttingen: Vandenhoeck & Ruprecht.

[ref49] Streib H. Klein C. (Eds.). (2018). Xenosophia and Religion: Biographical and Statistical Paths for a Culture of Welcome. Cham, Switzerland: Springer.

[ref50] Streib H. Klein C. Hood R. W. Jr. (2016). “Personality dimensions and versions of "spirituality"” in Semantics and Psychology of Spirituality: A Cross-cultural Analysis. eds. Streib H. Hood R. W. (Cham, Switzerland: Springer), 189–203.

[ref51] Streib H. Klein C. Keller B. Hood R. W. Jr. (2021). “The mysticism scale as measure for subjective spirituality: new results with Hood’s M-scale and the development of a short form” in Assessing Spirituality in a Diverse World. eds. Ai A. L. Harris K. A. Paloutzian R. F. Wink P. (Cham, Switzerland: Springer), 467–491.

[ref52] Tillich P. (1957). Dynamics of Faith. New York, NY: Harper & Row.

[ref53] Tong Y. P. Yang F. G. (2018). Internal diversity among "spiritual but not religious" adolescents in the United States: a person-centered examination using latent class analysis. Rev. Relig. Res. 60, 435–453. doi: 10.1007/s13644-018-0350-9

[ref54] Upenieks L. Ford-Robertson J. (2022). Changes in spiritual but not religious identity and well-being in emerging adulthood in the United States: pathways to health sameness? J. Relig. Health 61, 4635–4673. doi: 10.1007/s10943-022-01540-6, PMID: 35301635

[ref55] VanderWeele T. J. Mathur M. B. Chen Y. (2020). Outcome-wide longitudinal designs for causal inference: a new template for empirical studies. Stat. Sci. 35, 437–466. doi: 10.1214/19-STS728

[ref56] Vittengl J. R. (2018). A lonely search?: risk for depression when spirituality exceeds religiosity. J. Nerv. Ment. Dis. 206, 386–389. doi: 10.1097/NMD.0000000000000815 29652773

[ref57] Willard A. K. Norenzayan A. (2017). “Spiritual but not religious”: cognition, schizotypy, and conversion in alternative beliefs. Cognition 165, 137–146. doi: 10.1016/j.cognition.2017.05.018, PMID: 28544975

[ref58] Wink P. Ciciolla L. Dillon M. Tracy A. (2007). Religiousness, spiritual seeking, and personality: findings from a longitudinal study. J. Pers. 75, 1051–1070. doi: 10.1111/j.1467-6494.2007.00466.x, PMID: 17760857

[ref59] Wixwat M. Saucier G. (2021). Being spiritual but not religious. Curr. Opin. Psychol. 40, 121–125. doi: 10.1016/j.copsyc.2020.09.003 33069980

[ref60] Wong P. T. P. Cowden R. G. (in press). Accelerating the science and practice of psychology beyond WEIRD biases: enriching the landscape through Asian psychology. Front. Psychol. doi: 10.3389/fpsyg.2022.1054519 PMC9815563 36619071

[ref61] Zinnbauer B. J. Pargament K. I. Cole B. Rye M. S. Butter E. M. Belavich T. G.. (1997). Religion and spirituality: unfuzzying the fuzzy. J. Sci. Study Relig. 36, 549–564. doi: 10.2307/1387689

