# Supplementary material for: More spiritual than religious: Concurrent and longitudinal relations with personality traits, mystical experiences, and other individual characteristics
Source: Front Psychol. 2023 Jan 4;13:1025938. doi: 10.3389/fpsyg.2022.1025938 (PMC9846486; doi:10.3389/fpsyg.2022.1025938)
Supplement: Supplementary file 1 [file Data_Sheet_1.PDF]

More Spiritual than Religious: Concurrent and Longitudinal Relations with Personality Traits, Mystical Experiences, and Other Individual Characteristics

SUPPLEMENTAL FILE

Supplemental Text 1

An English version of the Wave 3 survey is available on the Open Science Framework at https://osf.io/64dcu/. A summary of the measures that were used to assess each outcome can be found in Supplemental Table S1. Means, standard deviations, and internal consistency estimates for all outcomes are reported in Supplemental Table S2.

Big Five Personality Traits

Participants completed the NEO Five Factor Inventory (NEO-FFI; Costa & McCrae, 1985). The 60-item measure assesses the Big Five personality traits (12 items each): neuroticism (e.g., "At times I have been so ashamed I just wanted to hide"), extraversion (e.g., "I really enjoy talking to people"), openness to experience (e.g., "I have a lot of intellectual curiosity"), agreeableness (e.g., "I would rather cooperate with others than compete with them"), and conscientiousness (e.g., "I try to perform all the tasks assigned to me conscientiously"). Participants responded to the items using a five-point scale ranging from 1 (*Strongly disagree*) to 5 (*Strongly agree*). This Five Factor Model of personality is one of the most cross-culturally studied psychological constructs. Contributions to the literature in personality psychology demonstrate cross-cultural validity as, for example, the reviews by McCrae (2002) and colleagues (McCrae et al., 2004) show. Also, norm values for various countries are available (Costa & McCrae, 1985; Egan et al., 2000; Körner et al., 2002; McCrae et al., 2005).

Psychological Well-being

We administered the Psychological Well-Being and Growth Scale (Ryff, 2010; Ryff & Singer, 1996). The measure assesses six dimensions of psychological well-being (7 items each): autonomy (e.g., "My decisions are not usually influenced by what everyone else is doing"), environmental mastery (e.g., "In general, I feel I am in charge of the situation in which I live"), positive relations with others (e.g., "I know that I can trust my friends, and

they know they can trust me"), personal growth (e.g., "I have the sense that I have developed a lot as a person over time"), purpose in life (e.g., "Some people wander aimlessly through life, but I am not one of them") and self-acceptance (e.g., "When I look at the story of my life, I am pleased with how things have turned out"). Participants responded to the items using a five-point scale ranging from 1 (*Strongly disagree*) to 5 (*Strongly agree*). Ryff's scale is one of the most widely used psychological measurements of well-being. It was included in major US surveys such as the Midlife in the United States (MIDUS) (Ryff, 2010), the National Survey of Families and Households II, and the Wisconsin Longitudinal Study (WLS), but also used in many other countries such as Chile (Urzúa et al., 2019; Cobo-Rendon et al., 2020), Colombia (Garzón-Velandia et al., 2020; Salas-Picón & Avendaño-Prieto, 2022), or China (for a review, see Zhou et al., 2022); and there are studies including cross-cultural comparison (Avsec et al., 2015; for a review, see Ruini & Cesetti, 2019).

Generativity

Participants responded to the 20-item Loyola Generativity Scale (LGS; McAdams & de St. Aubin, 1992), which measures the extent to which an individual has concern for the next generation (e.g., "I have made and created things that have had an impact on other people"). The items are rated on a four-point scale ranging from 1 (*Never applies to me*) to 4 (*Applies to me very often or nearly always*). A study in Cameroon, Costa Rica and Germany (Hofer et al., 2008) has used the LGS in cross-cultural comparison.

Mystical Experiences

We administered the Mysticism Scale (Hood, 1975). The measure contains three subscales that capture the core facets of a mystical experience: introvertive, which is composed of ego loss, timelessness/spacelessness, and ineffability, denoting an inward unitary consciousness beyond time and space (e.g., "I have had an experience that was both timeless

and spaceless"); extrovertive, which is framed by unity and inner subjectivity, implying an outward merging with the wholeness of all existence (e.g., "I have had an experience in which all things seemed to be aware"); and interpretative, which incorporates positive affect, sacredness, and noetic quality that qualify both types of mysticism (e.g., "I have had an experience in which a new view of reality was revealed to me"). Items are rated on a five-point scale ranging from 1 (*Very inaccurate*) to 5 (*Very accurate*). Mystical experiences, measured with Hood's (1975) mysticism scale, has been widely studied and validated across samples including Christians, Hindus, Tibetan Buddhists, Chinese Buddhists, Muslims in Malaysia, Pakistan, Iran, and Turkey (Anthony et al., 2010; Chen, Hood, et al., 2011; Chen, Qi, et al., 2011; Chen et al., 2012; Chen et al., 2013; Hood & Chen, 2013; Watson et al., 2015; Klein et al., 2016).

Religious Schemata

Participants completed the 15-item Religious Schema Scale (RSS; Streib et al., 2010). The measure includes three subscales (5 items each): truth of texts & teachings, a schema that features an exclusivist and authoritative understanding of one's own sacred texts (e.g., "What the texts and stories of my religion tell me is absolutely true and must not be changed"); xenosophia/inter-religious dialog, a schema reflecting an appreciation of difference, of the other, and of dialog (e.g., "We need to look beyond the denominational and religious differences to find the ultimate reality"); and fairness, tolerance & rational choice, a schema that features rational exchange of arguments (e.g., "We should resolve differences in how people appear to each other through fair and just discussion"). Items were rated on a five-point scale ranging from 1 (*Definitely not true*) to 5 (*Definitely true*). The RSS has been used in various cultures including, but not limited to, China, Germany, Greece, Malaysia, Indonesia, Iran, Pakistan, and USA (Streib et al., 2009; Kamble et al., 2014; Streib & Klein, 2014; Proios, 2015; Tekke et al., 2015; Ghorbani et al., 2016; Streib et al., 2016; Budiarto,

2017; Proios et al., 2017; Melles & Frey, 2018; Streib & Klein, 2018; Ardi et al., 2021; Walker & Rhoades, 2022; Palitsky et al., 2022).

Religiosity and Spirituality

Participants responded to the questions, “How religious are you?” and “How spiritual are you?” on five-point scales ranging from 1 (*Not religious*) to 5 (*Very religious*) and 1 (*Not spiritual*) to 5 (*Very spiritual*), respectively.

Religious Centrality

Participants responded to the seven-item Centrality of Religiosity Scale (CRS; Huber, 2009; Huber & Huber, 2012), which measures five dimensions of religiosity: public practice (“How often do you take part in religious services?”); private practice (e.g., “How often do you pray?”); religious experience (e.g., “How often do you experience situations in which you have the feeling that God or something divine intervenes in your life?”); ideology (“To what extent do you believe that God or something divine exists?“); and intellect (“How often do you think about religious issues?”). To ensure inclusiveness of non-Abrahamic religions, there are two items for the dimensions of private practice (“How often do you meditate?”) and experience (“How often do you experience situations in which you have the feeling that your are in one with all?”). For each of those dimensions, the item that receives a higher level of endorsement is used to calculate a total centrality of religiosity score. Items are rated using a five-point response scale ranging from 1 (*Never*) to 5 (*Very often*). The CRS has been used in the Religion Monitor, a large-scale cross-cultural survey including 21 countries in the 2008 survey (Bertelsmann, 2009) and 13 countries in 2013 (Bertelsmann & Pickel, 2013); thus, the CRS can be used for cross-religious comparison (Huber et al., 2020).

God Representation

We administered a 20-item version of the God Representation Scale (Silverman et al., 2016; Johnson et al., 2018), which assesses four different God representations using five adjectives each: the authoritarian God (i.e., strict, punishing, wrathful, stern, commanding);

the benevolent God (i.e., forgiving, merciful, compassionate, gracious, tolerant); the mystical God (i.e., nature, the universe, energy, consciousness, cosmic); and the ineffable God (i.e., unknown, unimaginable, unknowable, incomprehensible, inconceivable). A seven-point response scale ranging from 1 (*Strongly disagree*) to 7 (*Strongly agree*) was used to rate each item.

Intolerance of Ambiguity

Participants completed Budner’s (1962) Intolerance of Ambiguity Scale (IAS), which assesses the inclination to perceive ambigous situations as desirable rather than as sources of threat (e.g., “What we are used to is always preferable to what is unfamiliar”). Items are rated using a seven-point response scale ranging from 1 (*Strongly disagree*) to 7 (*Strongly agree*). The IAS is among the frequently used measures for intolerance of ambiguity and related constructs (for a review, see Furnham & Marks, 2013).

Need for Cognition

We administered the 18-item Need for Cognition Scale (NfC; Cacioppo et al., 1984). The measure assesses the openness and inclination to engage in thinking and reflection (e.g., “I really enjoy a task that involves coming up with new solutions to problems”). A seven-point response scale ranging from 1 (*Strongly disagree*) to 7 (*Strongly agree*) is used to rate each item. The NfC is used widely and also cross-culturally in countries such as China (Hui et al., 2022), Spain (Loose et al., 2022), Netherlands (Kramer et al., 2021; Schwaba & Bleidorn, 2021; Zainal & Newman, 2022), Indonesia (Sjabadhyni et al., 2021), France (Nicolas & Agnieszka, 2021), United Kingdom and USA (Lins de Holanda Coelho et al., 2020).

Supplemental Table S1

Measures Used to Assess Outcomes (Wave 1 to Wave 3)

Construct	Measure	Wave 1	Wave 2	Wave 3
Big Five personality traits	NEO Five Factor Inventory (Costa & McCrae, 1985)	x	x	x
Psychological well-being	Psychological Well-being and Growth Scale (Ryff, 2010)	x	x	x
Generativity	Loyola Generativity Scale (McAdams & de St. Aubin, 1992)	x	x	x
Mystical experiences	Mysticism Scale (Hood, 1975; Streib et al., 2021)	x	x	x
Religious schemata	Religious Schema Scale (Streib et al., 2010)	x	x	x
Religiosity	-	x	x	x
Spirituality	-	x	x	x
Religious centrality	(Huber & Huber, 2012)		x	x
God representation	God Representation Scale (Silverman et al., 2016; Johnson et al., 2018)			x
Intolerance of ambiguity	Intolerance of Ambiguity Scale (Budner, 1962)		x	x
Need for cognition	Need for Cognition Scale (Cacioppo et al., 1984)		x	x

Supplemental Table S2

Means, Standard Deviations, and Internal Consistency Estimates for All Variables

Variable	Study 1			Study 2 (Wave 1)			Study 2 (Wave 2)			Study 2 (Wave 3)		
	M	*SD*	α	*M*	*SD*	α	*M*	*SD*	α	*M*	*SD*	α
Big Five personality traits (range: 1-5)												
Neuroticism	2.69	0.68	.87	2.52	0.74	.87	2.70	0.73	.89	2.54	0.70	.89
Extraversion	3.41	0.55	.81	3.36	0.57	.81	3.23	0.56	.80	3.27	0.58	.80
Openness to experience	3.56	0.58	.79	3.96	0.46	.79	3.82	0.52	.73	4.04	0.47	.73
Agreeableness	3.69	0.49	.76	3.76	0.47	.76	3.70	0.49	.75	3.85	0.47	.80
Conscientiousness	3.65	0.55	.83	3.65	0.58	.83	3.58	0.57	.83	3.69	0.60	.84
Psychological well-being (range: 1-5)												
Autonomy	3.66	0.58	.70	3.79	0.55	.70	3.31	0.51	.45	3.28	0.50	.61
Environmental mastery	3.54	0.63	.78	3.65	0.69	.78	3.46	0.66	.79	3.61	0.68	.80
Personal growth	4.08	0.53	.76	4.31	0.48	.76	3.99	0.61	.77	4.28	0.49	.70
Positive relations with others	3.90	0.61	.77	3.94	0.62	.77	3.79	0.64	.76	3.95	0.63	.75
Purpose in life	3.80	0.59	.73	3.78	0.62	.73	3.63	0.64	.71	3.75	0.63	.70
Self-acceptance	3.69	0.63	.83	3.87	0.70	.83	3.63	0.69	.84	3.91	0.64	.85
Generativity (range: 1-4)	2.91	0.43	.85	2.94	0.43	.85	2.76	0.43	.84	2.88	0.47	.89
Mystical experiences (range: 1-5)												
Introvertive	3.47	0.99	.90	3.49	1.12	.90	3.26	1.02	.90	3.35	1.06	.89
Extrovertive	3.33	1.08	.90	3.28	1.25	.90	3.03	1.12	.90	3.06	1.28	.90
Interpretative	3.75	0.86	.88	3.69	0.98	.88	3.40	0.95	.89	3.48	0.99	.89
Religious schemata (range: 1-5)												
Truth of texts & teachings	2.79	1.17	.91	2.19	1.10	.91	2.33	1.16	.90	2.28	1.13	.91
Fairness, tolerance, & rational choice	4.12	0.63	.68	4.38	0.44	.68	4.28	0.57	.72	4.57	0.39	.60
Xenosophia/inter-religious dialog	3.38	0.79	.74	3.62	0.89	.74	3.38	0.82	.69	3.61	0.80	.67
Religiosity (range: 1-5)	2.50	1.42	-	2.33	1.56	-	2.44	1.39	-	2.57	1.54	-
Spirituality (range: 1-5)	3.28	1.50	-	3.56	1.58	-	2.99	1.39	-	3.19	1.52	-
Religious centrality (range: 1-5)							3.03	1.15	.89	3.20	1.09	.86
God representation (range: 1-7)												
Authoritarian										3.11	1.69	.91
Benevolent										4.34	1.90	.93
Mystical										4.45	1.75	.86
Ineffable										4.37	1.56	.81
Cognition (range: 1-7)												
Intolerance of ambiguity							3.47	0.59	.63	3.19	0.65	.60
Need for cognition							4.70	0.72	.82	4.79	0.76	.84

Note. *M* = mean, *SD* = standard deviation, α = alpha estimate of internal consistency (based on all available Study 1 and Study 2 data combined).

References

Anthony, F.-V., Hermans, C. A. M., & Sterkens, C. (2010). A comparative study of mystical experience among Christian, Muslim, and Hindu students in Tamil Nadu, India. *Journal for the Scientific Study of Religion*, *49*(2), 264-277.

Ardi, R., Tobing, D. H., Agustina, G. N., Iswahyudi, A. F., & Budiarti, D. (2021). Religious schema and tolerance towards alienated groups in Indonesia. *Heliyon*, *7*(7), e07603.

Avsec, A., Kavčič, T., & Jarden, A. (2015). Synergistic paths to happiness: Findings from seven countries. *Journal of Happiness Studies*, *17*(4), 1371-1390. https://doi.org/10.1007/s10902-015-9648-2

Bertelsmann, F. (Ed.). (2009). *What the world believes. Analysis and commentary on the Religion Monitor 2008*. Verlag Bertelsmann Stiftung.

Bertelsmann, S., & Pickel, G. (2013). *Religionsmonitor - verstehen was verbindet. Religiosität im internationalen Vergleich*. Verlag Bertelsmann Stiftung.

Budiarto, Y. (2017). Criterion (predictive) validation study of religious scheme scale. *Jurnal Psikologi*, *15*(1), 31-39.

Budner, S. (1962). Intolerance of ambiguity as a personality variable. *Journal of Personality*, *30*(1), 29-50. https://doi.org/10.1111/j.1467-6494.1962.tb02303.x

Cacioppo, J. T., Petty, R. E., & Kao, C. F. (1984). The efficient asessment of need for cognition. *Journal of Personality Assessment*, *48*(3), 306-307. https://doi.org/10.1207/s15327752jpa4803_13

Chen, Z., Ghorbani, N., Watson, P. J., & Aghababaei, N. (2013). Muslim experiential religiousness and Muslim attitudes toward religion: Dissociation of experiential and attitudinal aspects of religiosity in Iran. *Studia Religiologica*, *46*(1), 35-44. https://doi.org/doi:10.4467/20844077SR.13.003.1224

Chen, Z., Hood, R. W., Jr., Yang, L., & Watson, P. J. (2011). Mystical experience among Tibetan Buddhists: The common core thesis revisited. *Journal for the Scientific Study of Religion*, *50*(2), 328-338. https://doi.org/10.1111/j.1468-5906.2011.01570.x

Chen, Z., Qi, W., Hood, R. W., Jr., & Watson, P. J. (2011). Common core thesis and qualitative and quantitative analysis of mysticism in Chinese Buddhist monks and nuns. *Journal for the Scientific Study of Religion*, *50*(4), 654-670. https://doi.org/10.1111/j.1468-5906.2011.01606.x

Chen, Z., Zhang, Y., Hood, R. W., Jr., & Watson, P. J. (2012). Mysticism in Chinese Christians and non-Christians: Measurement invariance of the mysticism scale and implications for the mean

differences. *International Journal for the Psychology of Religion*, *22*(2), 155-168. https://doi.org/10.1111/j.1468-5906.2011.01570.x

Cobo-Rendon, R., Lopez-Angulo, Y., Perez-Villalobos, M. V., & Diaz-Mujica, A. (2020). Perceived social support and its effects on changes in the affective and eudaimonic well-being of Chilean university students. *Frontiers in Psychology*, *11*, Article 590513. https://doi.org/10.3389/fpsyg.2020.590513

Costa, P. T., & McCrae, R. R. (1985). *Revised neo personality inventory (NEO PI-R) and neo five-factor-inventory (NEO-FFI). Professional manual*. Psychological Assessment Resources, 1992.

Egan, V., Deary, I., & Austin, E. J. (2000). The NEO-FFI: Emerging British norms and an item-level analysis suggest n, a and c are more reliable than o and e. *Personality and Individual Differences*, *29*, 907-920.

Furnham, A., & Marks, J. (2013). Tolerance of ambiguity: A review of the recent literature. *Psychology*, *04*(09), 717-728. https://doi.org/10.4236/psych.2013.49102

Garzón-Velandia, D. C., Rozo, M. M., & Blanco, A. (2020). From war to life: Psychological well-being in adult victims of child recruitment. *Peace and Conflict: Journal of Peace Psychology*, *26*, 293-302. https://doi.org/10.1037/pac0000449

Ghorbani, N., Watson, P. J., Amirbeigi, M., & Chen, Z. (2016). Religious schema within a Muslim ideological surround: Religious and psychological adjustment in Iran. *Archive for the Psychology of Religion*, *38*(3), 253-277.

Hofer, J., Busch, H., Chasiotis, A., Kartner, J., & Campos, D. (2008). Concern for generativity and its relation to implicit pro-social power motivation, generative goals, and satisfaction with life: A cross-cultural investigation. *Journal of Personality*, *76*(1), 1-30. https://doi.org/10.1111/j.1467-6494.2007.00478.x

Hood, R. W., Jr., & Chen, Z. (2013). Mystical, spiritual, and religious experiences. In R. F. Paloutzian & C. L. Park (Eds.), *Handbook of the psychology of religion and spirituality, 2nd ed.* (pp. 422-440). The Guilford Press.

Huber, S. (2009). Religion Monitor 2008: Structuring principles, operational constructs, interpretive strategies. In F. Bertelsmann (Ed.), *What the world believes: Analysis and commentary on the Religion Monitor 2008* (pp. 17-51). Verlag Bertelsmann Stiftung.

Huber, S., Ackert, M., & Scheiblich, H. (2020). Religiosität in unterschiedlichen Religionskulturen – Vergleiche auf der Basis der Centrality of Religiosity Scale. *cultura & psyché*, *1*(1-2), 171-185. https://doi.org/10.1007/s43638-020-00007-3

Huber, S., & Huber, O. W. (2012). The Centrality of the Religiosity Scale (CRS). *Religions*, *3*(3), 710-724. https://doi.org/10.3390/rel3030710

Hui, Q., Yao, C., Huang, S., & You, X. (2022). Need for cognition and depressive symptoms: A mediation model of dysexecutive function and reappraisal. *Current Psychology*. https://doi.org/10.1007/s12144-021-02406-y

Johnson, K. A., Sharp, C. A., Okun, M. A., Shariff, A. F., & Cohen, A. B. (2018). SBNR Identity: The Role of impersonal God representations, individualistic spirituality, and dissimilarity with religious groups. *International Journal for the Psychology of Religion*, *28*(2), 121-140. https://doi.org/10.1080/10508619.2018.1416251

Kamble, S. V., Watson, P. J., Marigoudar, S., & Chen, Z. (2014). Varieties of openness and religious commitment in india: Relationships of attitudes toward Hinduism, Hindu religious reflection, and religious schema. *Archive for the Psychology of Religion*, *36*(2), 172-198. https://doi.org/doi 10.1163/15736121-12341283

Klein, C., Silver, C. F., Coleman, T. J., Streib, H., & Hood, R. W., Jr. (2016). "Spirituality" and mysticism. In H. Streib & R. W. Hood, Jr. (Eds.), *Semantics and psychology of "spirituality": A cross-cultural analysis* (pp. 165-187). Springer. https://doi.org/10.1007/978-3-319-21245-6_11

Körner, A., Geyder, M., & Brähler, E. (2002). Das Neo-fünf-Faktoren-Inventar: Validierung anhand einer deutschen Bevölkerungsstichprobe. *Diagnostica*, *48*(1), 19-27.

Kramer, A.-W., Van Duijvenvoorde, A. C. K., Krabbendam, L., & Huizenga, H. M. (2021). Individual differences in adolescents' willingness to invest cognitive effort: Relation to need for cognition, motivation and cognitive capacity. *Cognitive Development*, *57*, 100978. https://doi.org/10.1016/j.cogdev.2020.100978

Lins de Holanda Coelho, G., Hanel, P. H. P., & Wolf, L. J. (2020). The very efficient assessment of need for cognition: Developing a six-item version. *Assessment*, *27*(8), 1870-1885. https://doi.org/10.1177/1073191118793208

Loose, T., Vasquez-Echeverría, A., & Álvarez-Núñez, L. (2022). Spanish version of need for cognition scale: Evidence of reliability, validity and factorial invariance of the very efficient short-form. *Current Psychology*. Advance online publication. https://doi.org/10.1007/s12144-022-02739-2

McAdams, D. P., & de St Aubin, E. D. (1992). A theory of generativity and its assessment through self-report, behavioral acts, and narrative themes in autobiography. *Journal of Adult Development*, *62*, 1003-1015. https://doi.org/10.1037/0022-3514.62.6.1003

McCrae, R. R. (2002). NEO-PI-R data from 36 cultures. In R. R. McCrae & J. Allik (Eds.), *The five-factor model of personality across cultures* (pp. 105-125). Springer. https://doi.org/10.1007/978-1-4615-0763-5_6

McCrae, R. R., Costa, P. T., Martin, T. A., Oryol, V. E., Rukavishnikov, A. A., Senin, I. G., Hrebickova, M., & Urbanek, T. (2004). Consensual validation of personality traits across

cultures. *Journal of Research in Personality*, *38*(2), 179-201. https://doi.org/10.1016/s0092-6566(03)00056-4

McCrae, R. R., Martin, T. A., & Costa, P. T. (2005). Age trends and age norms for the neo personality inventory–3 in adolescents and adults. *Assessment*, *12*(4), 363-373.

Melles, E. A., & Frey, L. L. (2018). Promoting religious acceptance: The relationship between intercultural competence and religious attitudes among third culture kids. *Mental Health, Religion & Culture*, *20*(8), 812-826. https://doi.org/10.1080/13674676.2017.1413642

Nicolas, S., & Agnieszka, W. (2021). The personality of anthropomorphism: How the need for cognition and the need for closure define attitudes and anthropomorphic attributions toward robots. *Computers in Human Behavior*, *122*, 106841. https://doi.org/10.1016/j.chb.2021.106841

Palitsky, R., Kaplan, D. M., Brener, S. A., Mascaro, J. S., Mehl, M. R., & Sullivan, D. (2022). Do worldviews matter for implementation-relevant responses to mindfulness-based interventions? An empirical investigation of existential and religious perspectives. *Mindfulness*. Advance online publication. https://doi.org/10.1007/s12671-022-02010-6

Proios, M. (2015). Structure validity of the Religious Schema Scale in Greek. *Journal of Social Science Studies*, *2*(1), 343-353. https://doi.org/doi:10.5296/jsss.v2i1.6844

Proios, M., Dianni, M., Samara, E., & Syropoulos, D. (2017). Religiosity: Development of religious cognitive schemas and religious faith. *International Journal of Research Studies in Psychology*, *6*(2), 73-83. https://doi.org/10.5861/ijrsp.2017.1840

Ruini, C., & Cesetti, G. (2019). Spotlight on eudaimonia and depression. A systematic review of the literature over the past 5 years. *Psychological Research and Behavior Management*, *12*, 767-792. https://doi.org/10.2147/PRBM.S178255

Ryff, C. D. (2010). *Documentation of psychosocial constructs and composite variables in MIDUS II Project 1*. University of Wisconsin, Institute of Aging.

Ryff, C. D., & Singer, B. H. (1996). Psychological well-being: Meaning, measurement, and implications for psychotherapy research. *Psychotherapy and Psychosomatics*, *65*(1), 14-23. https://doi.org/10.1159/000289026

Salas-Picón, W. M., & Avendaño-Prieto, B. L. (2022). Adaptación de la escala de bienestar psicológico de ryff con una muestra de sobrevivientes del conflicto armado colombiano. *Revista Criminalidad*, *63*(3), 229-244. https://doi.org/10.47741/17943108.307

Schwaba, T., & Bleidorn, W. (2021). Internet use and cognitive engagement in older adulthood. *Social Psychological and Personality Science*, *13*(5), 968-977. https://doi.org/10.1177/19485506211049657

Sjabadhyni, B., Dwi Mustika, M., Carissa, N., Khairunnisa, S., Kurniawan, L. A., Mutiara, A. B., Putriaji, R., & Sinaga, A. P. (2021). The role of need for cognition in predicting the attitudes

of Indonesian millennials toward printed advertising and purchase intentions. *Sage Open*, *11*(3). https://doi.org/10.1177/21582440211029918

Silverman, G. S., Johnson, K. A., & Cohen, A. B. (2016). To believe or not to believe, that is not the question: The complexity of Jewish beliefs about God. *Psychology of Religion and Spirituality*, *8*(2), 119-130. https://doi.org/10.1037/rel0000065

Streib, H., Hood, R. W., Jr., & Klein, C. (2010). The Religious Schema Scale: Construction and initial validation of a quantitative measure for religious styles. *International Journal for the Psychology of Religion*, *20*(3), 151-172. https://doi.org/10.1080/10508619.2010.481223

Streib, H., Hood, R. W., Jr., Keller, B., Csöff, R.-M., & Silver, C. (2009). *Deconversion. Qualitative and quantitative results from cross-cultural research in Germany and the United States of America*. Vandenhoeck & Ruprecht. https://doi.org/10.13109/9783666604393

Streib, H., & Klein, C. (2014). Religious styles predict inter-religious prejudice: A study of German adolescents with the Religious Schema Scale. *International Journal for the Psychology of Religion*, *24*(2), 151-163. https://doi.org/10.1080/10508619.2013.808869

Streib, H., & Klein, C. (2018). Explaining xenophobia and xenosophia: Effects of religiosity, openness, tolerance of complexity, and religious schemata. In H. Streib & C. Klein (Eds.), *Xenosophia and religion: Biographical and statistical paths for a culture of welcome* (pp. 181-201). Springer. https://doi.org/10.1007/978-3-319-74564-0_6

Streib, H., Klein, C., & Hood, R. W., Jr. (2016). Religious schemata and "spirituality". In H. Streib & R. W. Hood, Jr. (Eds.), *Semantics and psychology of "spirituality": A cross-cultural analysis* (pp. 205-218). Springer. https://doi.org/10.1007/978-3-319-21245-6_13

Streib, H., Klein, C., Keller, B., & Hood, R. W., Jr. (2021). The Mysticism Scale as measure for subjective spirituality: New results with Hood's M-Scale and the development of a short form. In A. L. Ai, K. A. Harris, R. F. Paloutzian, & P. Wink (Eds.), *Assessing spirituality in a diverse world* (pp. 467-491). Springer. https://doi.org/10.1007/978-3-030-52140-0_19

Tekke, M., Watson, P. J., Hisham Ismaeli, N. A., & Chen, Z. (2015). Muslim religious openness and *ilm*. Relationships with Islamic religious reflection, religious schema, and religious commitments in malaysia. *Archive for the Psychology of Religion*, *37*(3), 295-320. https://doi.org/ 10.1163/15736121-12341313

Urzúa, A., Leiva, J., & Caqueo-Urízar, A. (2019). Effect of positive social interaction on the psychological well-being in South American immigrants in Chile. *Journal of International Migration and Integration*, *21*(1), 295-306. https://doi.org/10.1007/s12134-019-00731-7

Walker, A. C., & Rhoades, M. G. (2022). An examination of religious schemas through the lens of self-determination theory. *Christian Higher Education*, *21*(3), 191-213. https://doi.org/10.1080/15363759.2021.1929565

Watson, P. J., Ghorbani, N., Vartanian, M., & Chen, Z. (2015). Religious Openness Hypothesis II. Religious reflection and orientations, mystical experience, and psychological openness of Christians in Iran. *Journal of Psychology and Christianity*, *34*(2), 114-124.

Zainal, N. H., & Newman, M. G. (2022). Curiosity helps: Growth in need for cognition bidirectionally predicts future reduction in anxiety and depression symptoms across 10 years. *Journal of Affective Disorders*, *296*, 642-652. https://doi.org/https://doi.org/10.1016/j.jad.2021.10.001

Zhou, K., Lu, L., Hu, L., & Wang, Y. (2022). Associations between two conceptualizations of materialism and subjective wellbeing in China: A meta-analysis of studies from 1998 to 2022. *Frontiers in Psychology*, *13*, Article 982172. https://doi.org/10.3389/fpsyg.2022.982172
